# Poly(lipoic acid)-Based Nanoparticles as Self-Organized, Biocompatible, and Corona-Free Nanovectors

**DOI:** 10.1021/acs.biomac.0c01321

**Published:** 2020-12-21

**Authors:** Jakub W. Trzciński, Lucía Morillas-Becerril, Sara Scarpa, Marco Tannorella, Francesco Muraca, Federico Rastrelli, Chiara Castellani, Marny Fedrigo, Annalisa Angelini, Regina Tavano, Emanuele Papini, Fabrizio Mancin

**Affiliations:** ‡ Dipartimento di Scienze Chimiche, Università di Padova, via Marzolo 1, Padova, I-35131, Italy; § Dipartimento di Scienze Biomediche, Università di Padova, via U. Bassi 58/B1, Padova, I-35131, Italy; ∥ Centre for Innovative Biotechnological Research-CRIBI, Università di Padova, via U. Bassi 58/B1, Padova, I-35131, Italy; ⊥ Patologia Cardiovascolare e Anatomia Patologica, Dipartimento di Scienze Cardio-Toraco-Vascolari e Sanità Pubblica, Università di Padova, via Giustiniani 2, Padova, I-35128, Italy

## Abstract

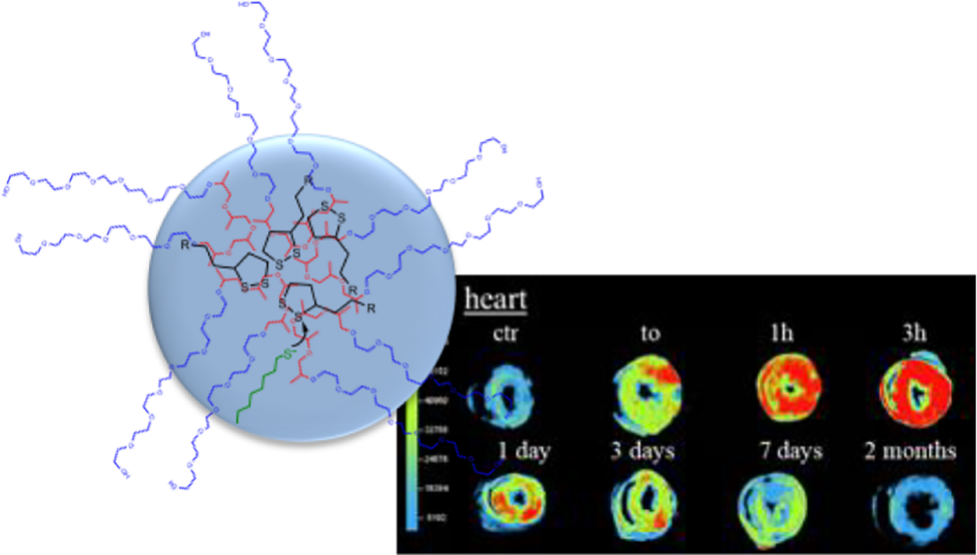

Herein we present an innovative approach to produce biocompatible, degradable, and stealth polymeric nanoparticles based on poly(lipoic acid), stabilized by a PEG-ended surfactant. Taking advantage of the well-known thiol-induced polymerization of lipoic acid, a universal and nontoxic nanovector consisted of a solid cross-linked polymeric matrix of lipoic acid monomers was prepared and loaded with active species with a one-step protocol. The biological studies demonstrated a high stability in biological media, the virtual absence of “protein” corona in biological fluids, the absence of acute toxicity in vitro and in vivo, complete clearance from the organism, and a relevant preference for short-term accumulation in the heart. All these features make these nanoparticles candidates as a promising tool for nanomedicine.

## Introduction

During the past decades, the use of nanoscale materials for therapeutic purposes has attracted wide interest for their relevant potential advantages.^1,2^ The possibility to introduce new therapeutic approaches, based on the peculiar properties of these materials,^3^ could help to overcome biological and metabolic obstacles and to deliver drugs precisely to a target site.^4^ In addition, nanosystems allow the combination of different modes of action to obtain multifunctional and smart systems.^5^ The development of nanomedicine agents is, however, encountering several obstacles that arise from the innate ability of living organisms to deal with and contrast nanosized entities.^6^ Formation of the protein corona, opsonization, mononuclear phagocyte system (MPS) capture, and physiological barriers are all examples of the difficulties that must be addressed.^7^ Additional problems arise from the long-term fate of the nanomedicine agents, that is, clearance, accumulation, degradation, and eventually toxicity. For these reasons, biodegradable polymer nanoparticles (NPs) represent an interesting approach.^8^ After their introduction in the organism, they slowly decompose, producing small nontoxic fragments that are easily excreted.^9^ The most successful example is represented by PLGA NPs.^10^ In this case, the nanoparticle matrix is an aggregate of copolymers of lactic and glycolic acids, which undergo hydrolytic cleavage by water and esterase enzymes.

Nanoparticle degradation, whose rate is essentially controlled by the monomers ratio, is usually accompanied by payload (drug) sustained release.^11,12^ However, several limitations have been encountered also with PLGA or similar nanoparticles. Their degradation starts as soon as they are exposed to water making difficult to control the release of the loaded drug. In addition, they must be stored in the dried form, and the resuspension process is sometimes problematic.^13^ Smart biodegradable nanocarriers stable in water and capable of releasing their payload only once they have reached the target are, hence, highly desirable.^14^ From this point of view, disulfide-based polymers represent an interesting solution. The disulfide bond is resistant to water, but is easily cleaved by thiols.^15^ Free thiols’ concentration is relatively low in extracellular fluids, but reaches significant values in cytoplasm (1–10 mM)^20,21^ due to the presence of the glutathione peptide.

Following such an approach, several groups have proposed organic and hybrid nanoparticles containing disulfide groups as degradation triggering agents.^16−19^ In particular, Matile and co-workers reported the synthesis of disulfide-based polymers via the Ring Opening Disulfide-Exchange Polymerization (RODEP)^22^ both for surface-functionalization and synthesis of cell-penetrating polymers. In this approach, cyclic disulfides, mainly lipoic acid derivatives, undergo thiolate-initiated polymerization. When they were functionalized with guanidinium residues,^22^ the resulting polycationic polymers spontaneously penetrate cell membranes, delivering dyes, drugs, proteins, and even quantum dots. In the cytoplasm, the polymer is cleaved very quickly by endogenous thiols.^15^ Subsequently, RODEP was used by Waymouth to induce cross-linking of block copolymers to form reversible gels^23^ and by Feringa and Qu to realize polymers capable of supramolecular networking.^24^ The reaction was further studied by J. Moore who described how to control the formation of linear or cyclic polymers^25^ and by Lu who used it to prepare protein–polydisulfide conjugates.^26,27^

Poly(lipoic acid) derivatives prepared by RODEP are ideal candidates for the formulation of biodegradable nanoparticles. Not only is lipoic acid an endogenous molecule, but it also has antioxidant and anticancer properties. For these reasons, it is used as a dietary supplement, to treat various diseases such as atherosclerosis, thrombosis and diabetes,^29,30^ and even, in selected cases, as active payload for organic or lipid nanoparticles.^15,31^ As an example, the groups of Chen and Gao formulated poly(lipoic acid) polymers into nanoparticles, using procedures similar to those typical of PLGA nanoparticles,^28,32^ and demonstrated that they are promising vectors for drug delivery accompanied by sustained release. Yu and We formulated nanoparticles made by a cyclodextrin-initiated cationic poly(lipoic acid) and RNA for the codelivery of nucleic acids and drugs.^33^

This paper describes a new step toward the validation of polylipoic nanoparticles as universal nanovectors for biomedical applications. We demonstrate that the flexibility of lipoic acid as a precursor is such that it makes possible the direct synthesis of cross-linked, dye-loaded, PEG-protected polylipoic nanoparticles in a single step starting from small molecule precursors. Advantages for large-scale production are evident. In addition, these nanoparticles proved to undergo degradation in the presence of thiols and revealed interesting biological properties in preliminary biocompatibility and biodistribution studies.

## Experimental Section

### Synthesis of the Precursors

Chemical reagents and solvents were purchased from Aldrich and used without further purification. Water was purified using a Milli-Q water purification system. Reactions were monitored by TLC on 0.25 mm Merck silica gel plates (60 F254).

NMR spectra were recorded on a AVIII 500 spectrometer (500 MHz for 1H frequency) or on a Bruker AC-300 (300 MHz for 1H frequency). UV–vis absorption spectra were measured on a Varian Cary 50 spectrophotometer with 1 cm path length quartz cuvettes. Fluorescence spectra were measured on a Varian Cary Eclipse fluorescence spectrophotometer. Both the spectrophotometers were equipped with thermostated cell holders. ESI-MS were recorded on an Agilent Technologies 1100 Series system equipped with a binary pump (G1312A) and MSD SL Trap mass spectrometer (G2445D SL).

Compounds **1**–**12** were synthesized by standard procedures (details in the Supporting Information) and fully characterized.

### Nanoparticles Synthesis

An acetone solution of the selected precursor or precursors (20 mL, 5 mg/mL) was added dropwise with a syringe pump (0.5 mL/min) to an aqueous solution (100 mL) of pluronic surfactant (100 mL, 20 mM) buffered at pH 7.4 with PBS (2 mM of 2 mM of phosphate buffer) under stirring (1000 rpm). After the addition of precursor, the solution was left stirring for 30 min. Subsequently, 2 mL of a solution of 1-octanethiol (5 mg/mL) in acetone was added and, after 90 min, 2 mL of a iodoacetamide (5 mg/mL) solution in acetone was added. The milky reaction mixture was concentrated under reduced pressure and filtered with a 0.20 μm cutoff cellulose acetate filter. Nanoparticles were collected by centrifugation and purified by resuspension in PBS (pH 7.4, 1 × 12 mL, 2 mM of phosphate buffer) and in H_2_O (2 × 12 mL each). The nanoparticles were stored at 4 °C as a water suspension. Stock solutions were used for the investigation described (lyophilization, degradation, biological experiments) upon dilution in the appropriate medium. The same procedure was used to prepare the PEG–PLGA nanoparticles.

The hydrodynamic particle size (dynamic light scattering, DLS) and *Z*-potential were measured with a Malvern Zetasizer Nano-S equipped with a HeNe laser (633 nm) and a Peltier thermostatic system. Measurements were performed at 25 °C in water or PBS 10 mM buffer at pH 7. Transmission electron microscopy (TEM) was recorded on a FEI Tecnai G12 microscope operating at 100 kV. The images were registered with a OSIS Veleta 4K camera. Thermogravimetric analysis (TGA) was run on 100 μL nanoparticle samples using a Q5000 IR instrument from 25 to 1000 °C under a continuous air flow. NMR spectra in the solid state were collected on a Varian 400 equipped with a narrow bore, triple resonance T3MAS probe spinning 4 mm rotors and operating at ^1^H and ^13^C frequencies of 400.36 and 100.68 MHz, respectively. The nominal temperature of the probe was always set to 298 K. ^13^C CP-MAS spectra were acquired at 5 kHz MAS with 1200 scans and a repetition delay up to 3 s. The contact time for CP was 2 ms, and an acquisition time of 50 ms was used.

### Determination of Serum or Plasma Proteins Associated with NPs

NPs (50 μg/mL) were incubated at 37 °C for 30 min with 10%, 20%, or 50% HS (or HP or FCS) diluted in RPMI-1640 medium, recovered by centrifugation (30 min, 12000 rpm at 4 °C), washed one or three times with PBS, and dissolved in 25 μL of loading sample buffer. NP pellets were heated at 95 °C for 5 min and loaded in equal volumes (12 μL) on a 12% (v/v) SDS-PAGE. Proteins were stained with the Silver Staining protocol or blotted onto PVDF membrane (Amersham) and HRG, HSA, or Apo A1 were detected by specific antibodies (Abnova for HRG, Calbiochem for HSS and Apo A1) by enhanced chemiluminescence reaction.

### Plasma Clotting Time

A total of 77 μL of HP were added to 100 μL of NPs or Ludox at various concentrations in 150 mM NaCl in a 96-well microtiter plate (Sarstedt), and coagulation was started by the addition of 23 μL of 150 mM CaCl_2_. The plate was incubated at 37 °C, and the changes in optic density were read at 405 nm every 60 s for 60 times. To calculate the mean absorbance at each time point, three wells were averaged per sample. The time required to reach half maximal absorbance increase (*t*_1/2_) was calculated and used for statistical analysis.

### C3a Detection

To control the complement activity, 25 μL of HS were treated with 6.25 μL of zymosan (25 mg/mL, Sigma, prepared as described by manufacturer’s instructions) for 30 min at 37 °C; the reaction was stopped with 25 mM EDTA. To assess the complement activation of HS induced by NP incubation, 25 μL of HS were incubated with different concentrations of NPs for 30 min at 37 °C. Then 1.6 μL of each sample were mixed with 38.4 μL of water and 6.7 μL of loading sample buffer, and 15 μL of sample were loaded onto a 12% gel. Proteins were then blotted onto a PVDF membrane, and C3a was detected by specific antibodies (Cabiochem) by enhanced chemiluminescence reaction.

### Hemolysis Assay

Human erythrocytes were obtained from the human blood of healthy volunteers after elimination of buffy coats, washed in PBS, and further treated with different NP doses (up to 100 μg/mL) in triplicate; after a 2 h incubation, samples were centrifuged (1500 rpm for 5 min) and supernatant absorbance was determined at 540 nm; data were expressed as a percentage with respect to positive control (human erythrocytes incubated with water).

### Cells

HeLa and Raw 264.7 cells were maintained in DMEM (Invitrogen), supplemented with 10% FCS (Euroclone) and antibiotics (penicillin and streptomycin, Invitrogen) at 37 °C in a humidified atmosphere containing 5% (v/v) CO_2_; cells were split every 2–3 days. Human macrophages were obtained from human monocytes, purified from buffy coats of healthy donors by means of two sequential centrifugations on Ficoll and Percoll (GE Healthcare) gradients, and differentiated for 7 days with 100 ng/mL macrophage colony-stimulating factor (M-CSF, BD Biosciences) in RPMI-1640 plus 20% FCS. Total human leukocytes were obtained from buffy coats after erythrocytes lysis by hypotonic shock in 155 mM NH_4_Cl, 10 mM KHCO_3_, and 100 mM Na_2_EDTA at pH 7.4 for 3 min at room temperature.

### MTT Assay

The day before the experiment, cells (HeLa, human leukocytes, human macrophages, and mouse Raw 264.7) were seeded onto 24-well plate (Falcon), as indicated in Figure 8B,C. The day of the experiment, cells were treated with different concentrations of NPs (up to 100 μg/mL, as indicated in Figure 8) in cellular medium supplement with 10% FCS; after 24 h or 6 days incubation, wells were sucked off and incubated with 100 μL of MTT (Promega) at 37 °C until color development; absorbance was red at 492 nm, and the percentage of alive cells was calculated with respect to nontreated cells.

### In Vivo Experiments

Health male Sprague–Dawley rats, weighing 200 g, were injected with 2 mg/rat of rhodamine red labeled F127@**7**-NPs via tail vein. Rats were housed in a temperature-controlled environment (21–22 °C) on a 12 h light/dark cycle, with access to water and food at all times.

At different time points (*t*_0_, 1 h, 3 h, 1 day, 3 days, 7 days, and 1/2 months) from injection, rats were randomly killed and blood and organs were collected. Organs have been removed in toto and washed in distilled water and fixed in formalin to reduce passive dissemination on the cut surface.

Experiments were approved by the University of Padua Ethical Committee and from Italian National Health Institute.

### Neutrophil Gelatinase-Associated Lipocalin (NGAL) Assessment on Sera

Neutrophil gelatinase-associated lipocalin (NGAL) was measured on sera with an enzyme-linked immunoassay (Rat NGAL ELISA kit; BIOPORTO Diagnostics, Bioexe Research Technology, Verona, Italy) following the manufacturer’s instructions. The antibody was specific for rat NGAL.

### NGAL Expression in the Heart Tissue

Heart samples were homogenized and solubilized in sodium dodecyl sulfate (SDS) buffer. Protein quantification was performed using Qubit Proteinassay Kit (Life Technologies, Monza, Italy) according to the manufacturer’s instructions. Protein samples were mixed with a nonreducing and reducing buffer and incubated for 5 min at either room temperature (reducing and nondenaturating conditions) or 95 °C (reducing and denaturating conditions). All samples were subsequently separated on a 10% gel in SDS-PAGE and transferred onto a nitrocellulose membrane (Amersham, Euroclone, Italy). The membrane was blocked for 1 h with 5% nonfat milk in the TBS containing 0.5% (v/v) Triton X-100. (Sigma-Aldrich) and incubated overnight with polyclonal goat antibodies against NGAL (1:500, Abcam, Prodotti Gianni, Milan, Italy). Blots were developed using the Super Signal West Femto ECL substrate (Pierce, Euroclone, Italy). Blot image acquisition was performed using Alliance 2.7 (UVITEC, Eppendorf, Italy) and software Alliance 2.7 1D fully automated.

### Organs Biodistribution of NPs

Slices of about 3–5 mm thickness of different organs were introduce in the Alliance 2.7 (UVITEC, Eppendorf, Italy). Rhodamine red-labeled F127@**7**-NPs fluorescence was obtained under chroma Alliance 2.7 and was analyzed by alliance 3D software (UVITEC, Eppendorf, Italy). The macrodistribution analysis of NPs fluorescence signal was obtained with the same gain and setup of the image analyzer. The intensity of the fluorescence is represented and normalized as a pseudocolor scale bar that is consistent for all images, and that is reported in the Figure 10A.

### Laser Scanning Confocal Microscopy Analysis and NP Localization

Fixed rat organs were dehydrated with ethanol, cleaned with xylene, embedded in paraffin, and sliced into 5 μm sections. One section for each organ was counterstained with TO-PRO-3 for nuclei identification (Invitrogen, Molecular Probes, Eugene, OR) following standard procedures. Micrographs were taken using a laser scanner confocal microscope (Model TCS-SL; Leica, Germany) equipped with beam splitter 488, 543, and 605 nm and FW TD 488/543/633 beam splitting excitation mirrors. Laser scanning confocal microscopy analysis was performed keeping acquisition parameters (laser power, aperture width, opening percentage, and gain) constant.

## Results and Discussion

### Lipoic Acid Derivatives as Nanoparticle Precursors

Several methods have been reported for the preparation of polymeric nanoparticles, including polylipoic ones.^28,32,34^ Most of them, however, involve the use of presynthesized polymers that are assembled to form nanoparticles. The eventual “stealth” coating, that is, surface functionalization with poly(ethylene glycol)^35^ or similar species,^36^ requires either pre- or postfunctionalization steps. In addition, cross-linking of the polymer chains, which should increase the stability of the nanoparticles, is not possible. In most of the cases (as with polyesters or polyamides), the direct synthesis of the nanoparticles with methods such as microemulsion polymerization is not possible because of the incompatibility of the polymer precursors or reaction catalysts with the aqueous medium.^37^

**Figure 1:**
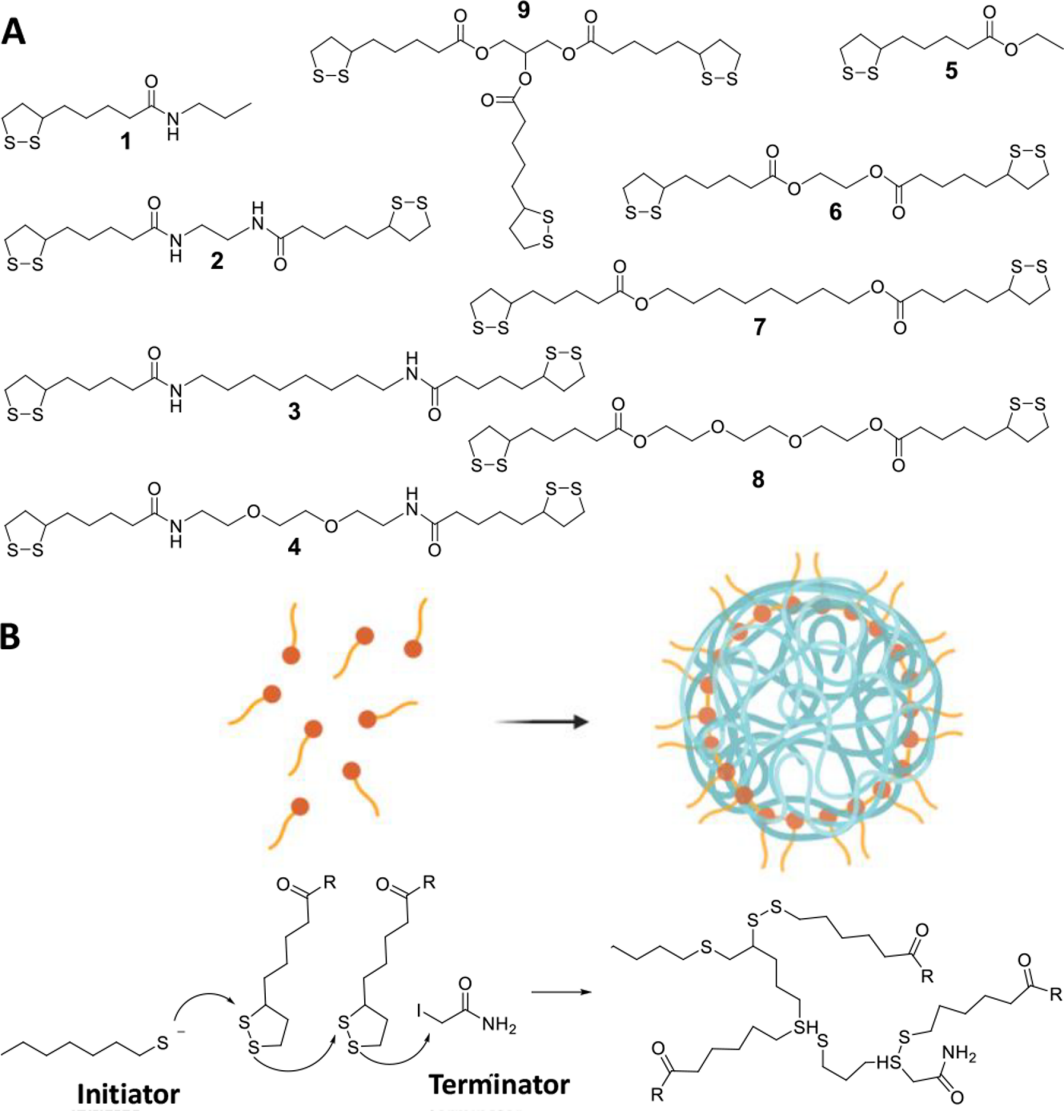
(A) Chemical structure of the lipoic acid derivatives (**1**–**9**) studied in this work. (B) Schematic representation of RODE polymerization inside pluronic-stabilized organic nanoparticles used to form polylipoic nanoparticles.

On the contrary, the RODEP reaction is fully compatible with the presence of water.^29,22,29^ In addition, Lee and co-workers^15^ have already demonstrated that nanoparticles made by lipophilic derivatives of lipoic acid can be prepared with the nanoprecipitation method.^38,39^ Based on these premises, we synthesized a set of lipoic acid derivatives (Figure 1) which, after selection, were used for the preparation of polymeric NPs. These included monofunctional derivatives **1** and **5**, presenting an ester and amide functional group, a series of bifunctional derivatives of lipoic acid linked by diamine (**2**–**4**) or diol (**6**–**8**) spacers, and the trifunctional derivative **9**, presenting glycerol as a tridentate spacer. Although some monomer precursors were not biocompatible, in particular, some diamines, their study allowed a deeper understanding of the structure–properties correlations controlling the nanoparticles’ formation and behavior.^37,38^

**Figure 2:**
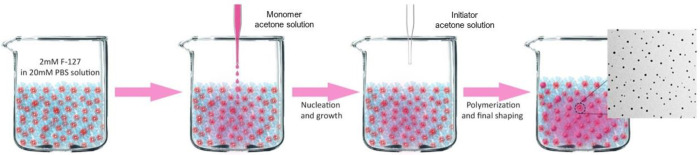
Nanoprecipitation method used to synthesize the nanoparticles. A solution of the precursor in acetone is added to an aqueous solution of surfactant (2 mM) and PBS buffer (pH 7.4, 20 mM). Upon mixing, acetone diffuses, and the precursor precipitates into the micelles, due to its hydrophobic character. Precursor polymerization is initiated by the addition of a small amount of octanethiol in acetone and terminated by the addition of iodoacetamide in acetone (not shown).

All the precursors were prepared in good yields by standard coupling protocols. As usual in the case of lipoic acid derivatives, they all showed the tendency to spontaneous polymerization. However, most of them could be stored at dark and low temperature both in the solid state and in solution for several weeks.^26^ Diamide derivatives **2** and **3** were poorly soluble in acetone, which is the solvent chosen for the preparation of nanoparticles.

### Nanoparticles’ Synthesis and Size Control

Nanoparticles made by lipoic acid derivatives were prepared according to the nanoprecipitation protocol (Figure 2). An acetone solution of the selected precursor (5 mg/mL) was injected into an aqueous solution of the neutral surfactant Pluronic F127 (1 mM), buffered at pH 7 with PBS, under vigorous stirring.^42^ The volume ratio between the organic and aqueous solutions was 1:10. Upon mixing, the diffusion of the organic solvent in water induced the immediate aggregation of the insoluble precursors and the formation of surfactant-stabilized organic nanoparticles. The RODEP polymerization of the lipoic acid derivatives was induced by the addition of a lipophilic thiol (1-octanethiol). Finally, the polymerization reaction, as well as the possible excess of 1-octanethiol, was quenched with 2-iodoacetamide (Figure 1).

**Figure 3:**
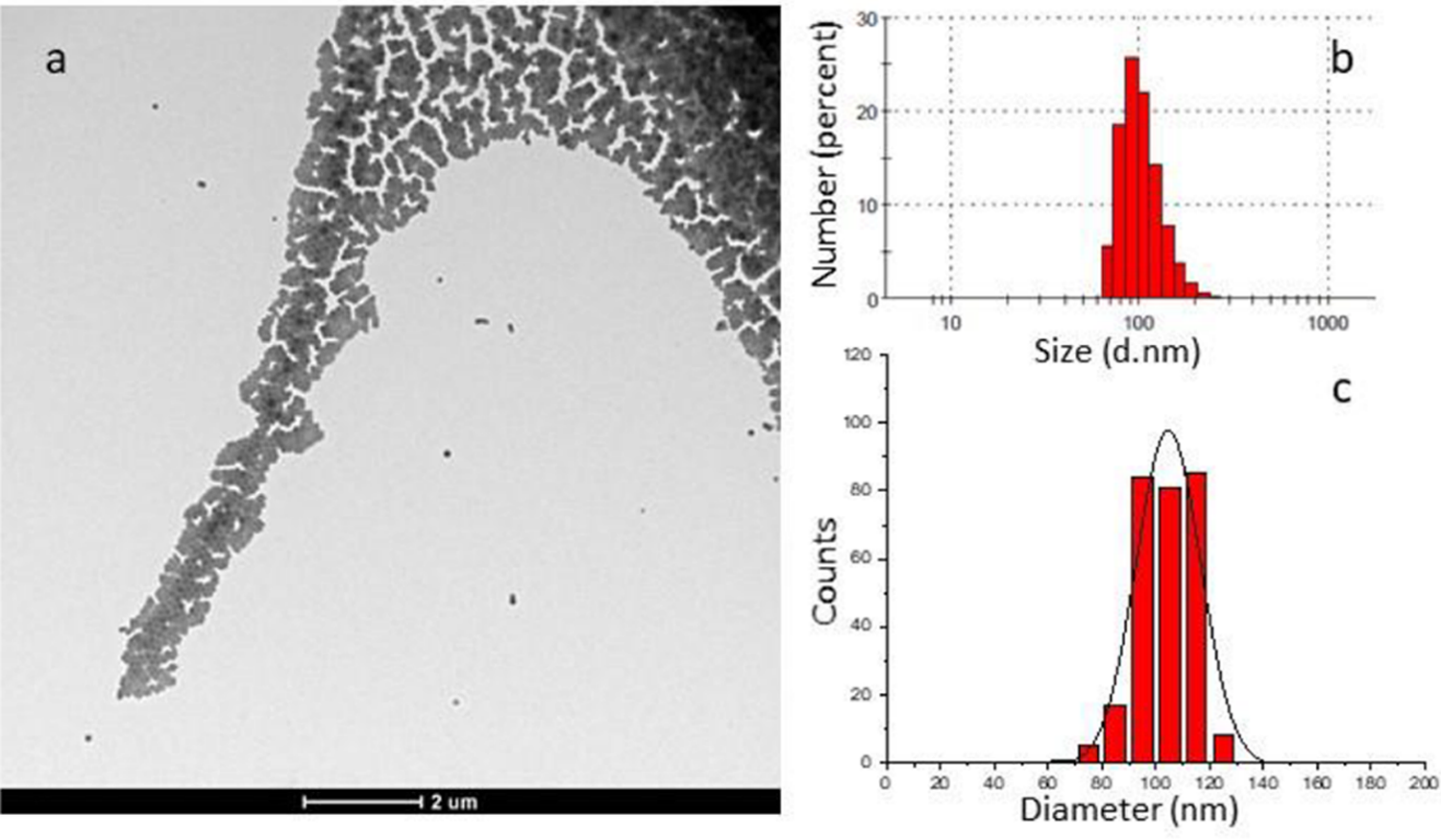
(A) TEM micrograph and (B) nanoparticle size distribution (number weighted) determined by DLS of F127@**9**-NPs (average size 102 nm, PDI: 0.088 measured by DLS). (C) Nanoparticle size distribution determined by TEM.

The formed polymeric NPs were purified by centrifugation and resuspended in water or PBS buffer. transmission electron microscopy and dynamic light scattering (DLS) analyses revealed average diameters ranging from 100 to 150 nm and a quite homogeneous size distribution (Figure 3). Similar results (see infra) were obtained with all the precursors tested. Larger NPs (average diameter of 200–300 nm) were obtained when the volume ratio of the organic and water solutions was increased to 1:1.

**Figure 4:**
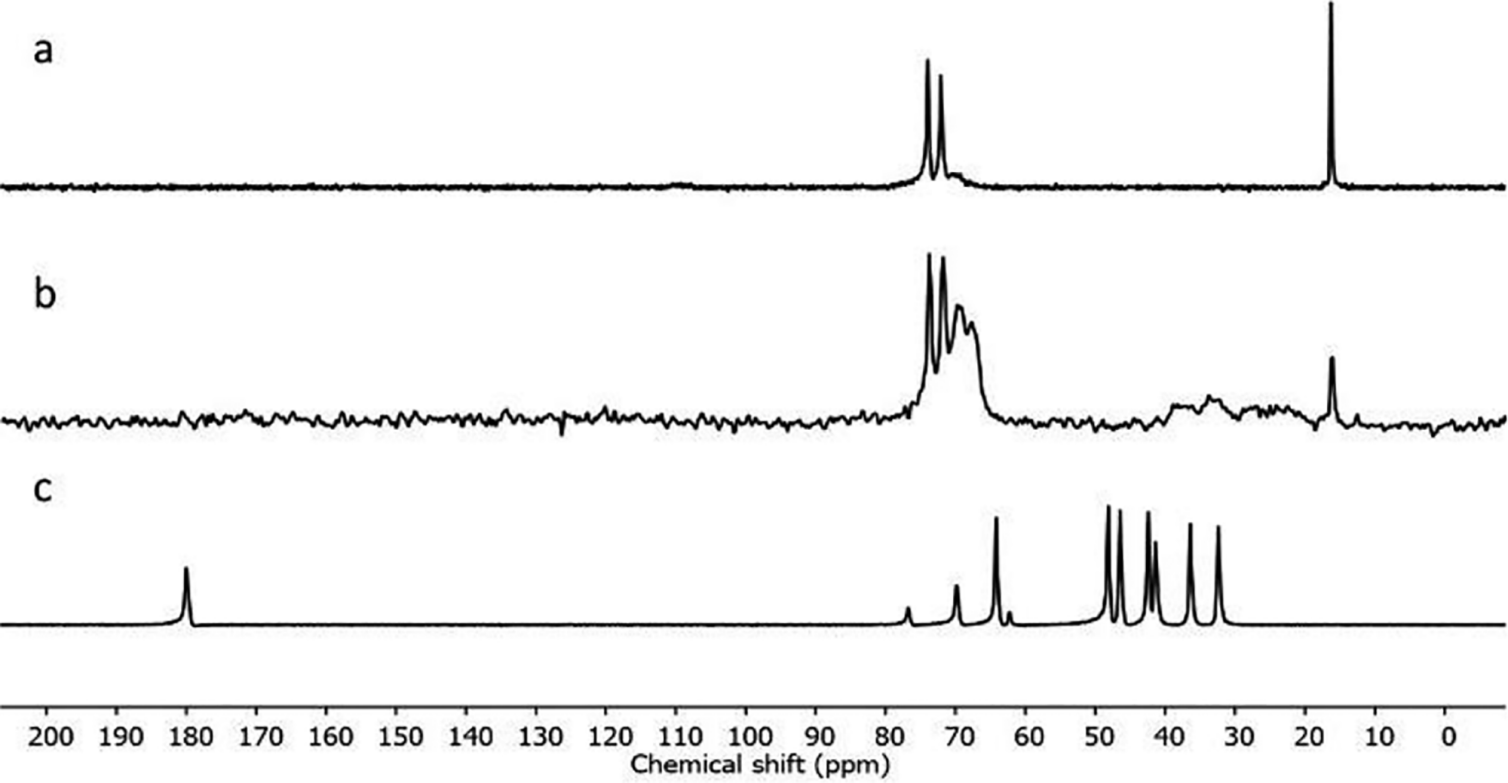
CP-MAS SS-^13^C-NMR of (a) pure F-127, (b) lyophilized F127@9-NPs (average size 132 nm, PDI: 0.082 measured by DLS), and (c) pure **9**.

The formation of cross-linked polylipoic NPs by RODEP polymerization was first confirmed by cross-polymerization magic angle spinning (CP-MAS) solid state ^13^C NMR. We compared the spectrum of a sample of dried NPs, prepared with precursor **9** and the F127 Pluronic surfactant (F127@**9**-NPs), with that of pure F127 and **9**. The NMR spectra of F127 (Figure 4a) and **9** (Figure 4c) showed sharp signals, as expected for compounds in the physical state of viscous liquids or gels. In the case of F127@**9**-NPs (Figure 4b), signals corresponding to the spins of monomer **9** were instead broad and substantially weaker. Signals relative to F127 remained sharp, but the intensity of the signal at 18 ppm is substantially lower than in the pure sample. Cross-polarization experiments enhance the signals of mobile moieties with respect to more rigid ones. Hence, the mobility of **9** residues in the nanoparticles sample was substantially reduced with respect to the surfactant molecules, suggesting the polymerization of the lipoic acid moieties in the particles’ cores. Also, the surfactant signals provided relevant information. F127 is a diblock copolymer composed by three fragments: two hydrophilic terminal poly(ethylene glycol) portions (PEG, 100 units each, PEG_4400_) and a hydrophobic poly(propylene glycol) inner portion (PPG, 65 units).^40^ Due to its structure, it adopts a U shape when it forms a micellar aggregate. The fact that the signal at 18 ppm, relative to the inner poly(propylene glycol) portion of F127, was less intense with respect to the pure sample suggests that this portion experiences at least partially a mobility decrease, with the consequent signal broadening. This evidence confirmed that the surfactant remained adsorbed on the nanoparticles’ surface or even entangled in the polymer matrix, providing the nanoparticles with a poly(ethylene)glycol coating. This should ensure “stealth” properties to the NPs, that is, the ability to avoid the absorption of plasma proteins and consequently escape macrophage capture.^46^ Moreover, Pluronic surfactants have been approved by the FDA as a safe and inert additive for medical formulations.^41^

It is relevant to note that lipoic acid derivatives can undergo polymerization even in the absence of initiators. Hence, to evaluate if precursors polymerization is the result of the addition of the thiol initiator, we compared the properties of F127@**9** nanoparticles produced with and without the addition of initiator and quencher. Size of the nanoparticles upon prolonged incubation in water was monitored with DLS (Table S2). In the case of nanoparticles produced with the initiator, no relevant variations of both size and polydispersity index (PDI) were detected after 21 days. On the other hand, nanoparticles prepared without the initiator underwent a relevant size increase, doubling the average hydrodynamic diameter in 21 days. In addition, PDI substantially increased. Swelling, as well as decreased electron density, was confirmed also by TEM analysis (Figure S18). The reasons for the observed swelling are still unclear, but it appears quite evident that the thiol-initiated RODEP led to the formation of more rigid and stable nanoparticles.

We could determine the amount of F127 bound to the nanoparticles by performing elemental analysis on several batches of F127@**7**-NPs and F127@**9**-NPs (Table S1). Using the sulfur content to separate the contribution of the precursors and the surfactant, we could determine that the F127 content was on average 22.6% (F127@**7**-NPs, 180 nm) and 28.5% (F127@**9**-NPs, 144 nm). Tentative calculations performed assuming the density of 0.6 g/cm^3^ for the polylipoic cores indicated a footprint of 4.1 ± 1.1 and 4.0 ± 1.6 nm^2^ respectively, and an average distance between the grafting points of 2.3 nm in both cases. The Flory radius of PEG_4400_ is 5.5 nm;^43^ consequently, the footprint values obtained indicate a brush PEG shell.^43^

Having established a general protocol to prepare cross-linked polydisulfide nanoparticles, we investigated the influence of the synthetic parameters on the particles size. First, we explored the role of the chemical structure of the pluronic surfactant used on the NPs assembly (Table 1). The preparation of NPs was repeated using F-68 (featuring a PEG/PPG/PEG ratio of 76–29–76) and P-123 (ratio of 20–70–20) and precursor **8**. DLS analysis showed that nanoparticles prepared with surfactants F-68 and F-127 had similar average sizes and distributions; meanwhile, the use of surfactant P-123 did not lead to the formation nanoparticles stable enough to survive the purification procedure. This suggested that a high PEG/PPG ratio in the surfactant is needed to efficiently stabilize the nanoparticles.

**Table 1:** Average Hydrodynamic Size and Dispersion (Expressed as Polydispersity Index, PDI) of Nanoparticles Prepared from Precursor **8** and Different Pluronic Surfactants

surfactant	size (nm)	PDI
F-68	115 ± 36.7	0.102
F-127	141 ± 42.3	0.090
P-123	26.4 ± 10.2	0.149

Surfactants F-68 and F-127 were then studied for the preparation of nanoparticles with the acetone-soluble precursors **5**, **7**, **8**, and **9** (Table 2 and Figures S10–S17), all featuring an ester group. In most of the cases, we obtained similar average diameters of the NPs and relatively narrow distributions (indicated by the polydispersity index, PDI) with the two surfactants.

**Table 2:** Average Hydrodynamic Size and Dispersion (Expressed As Polydispersity Index, PDI) of Nanoparticles Prepared from Precursors **5**–**9** and Different Pluronic Surfactants

monomer	surfactant	size (nm)	PDI
**5**	F-68	198 ± 84.2	0.181
	F-127	137 ± 41.6	0.092
**7**	F-68	122 ± 16.4	0.018
	F-127	121 ± 19.9	0.027
**8**	F-68	119 ± 34.1	0.082
	F-127	132 ± 44.8	0.115
**9**	F-68	96.9 ± 36.4	0.141
	F-127	91.2 ± 28.4	0.097

Noticeably, monofunctional precursor **5**, produced NPs with larger average size and broader distribution when used with F-68 as surfactant. This effect could be ascribed to the fact that monomer **5** cannot form a cross-linked polymeric network at difference from precursors **7**–**9**. However, we noticed that a small size trend is observed by moving from **5** to **9**, that is, by increasing the molecular weight and hydrophobicity of the precursors. In the case of organic nanoparticles prepared with similar precursors, it has been reported that size and hydrophobicity of the precursors may have an effect on the size of the nanoparticles leading to the formation of smaller particles in the nanoprecipitation phase.^29^ In our case, this effect, even if relatively modest, was confirmed, suggesting that precursor molecular weight is the main parameter affecting the final size of the nanoparticles.

The “amide” precursors **1**–**4** were not investigated in detail since only **1** and **4** were revealed to be soluble in acetone. However, preliminary experiments showed that precursor **8** behaved similar to precursor **5**.

We also studied the effect of the concentration of the precursor on the size of the nanoparticles prepared with monomers **7** and **9** and the surfactant F127 (Table 3). The results indicated a generally small dependence of the NP size from the precursors concentration. However, in the case of precursor **7**, larger particles were obtained at selected precursor concentrations (4 and 3 mg/mL). This suggests that other synthetic parameters not investigated here, in particular, the mixing efficiency, can be relevant in determining the size of the nanoparticles formed.^37,38^

**Table 3:** Average Hydrodynamic Size and Dispersion (Expressed As Polydispersity Index, PDI) of Nanoparticles Prepared from Precursors **8** and **9** and F127 Surfactant Using Different Precursor Concentrations

monomer	monomer concn (mg/mL)	size (nm)	PDI
**7**	5	127 ± 54.9	0.187
	4	166 ± 74.4	0.201
	3	162 ± 58.9	0.132
	2	129 ± 59.3	0.211
	1	136 ± 34.4	0.064
**9**	5	91.2 ± 28.4	0.097
	4	93.3 ± 33.4	0.128
	3	85.2 ± 29.8	0.122
	2	90.2 ± 30.7	0.116
	1	81.4 ± 29.1	0.128

### Fluorescent-Doped Nanoparticles

**Figure 5:**
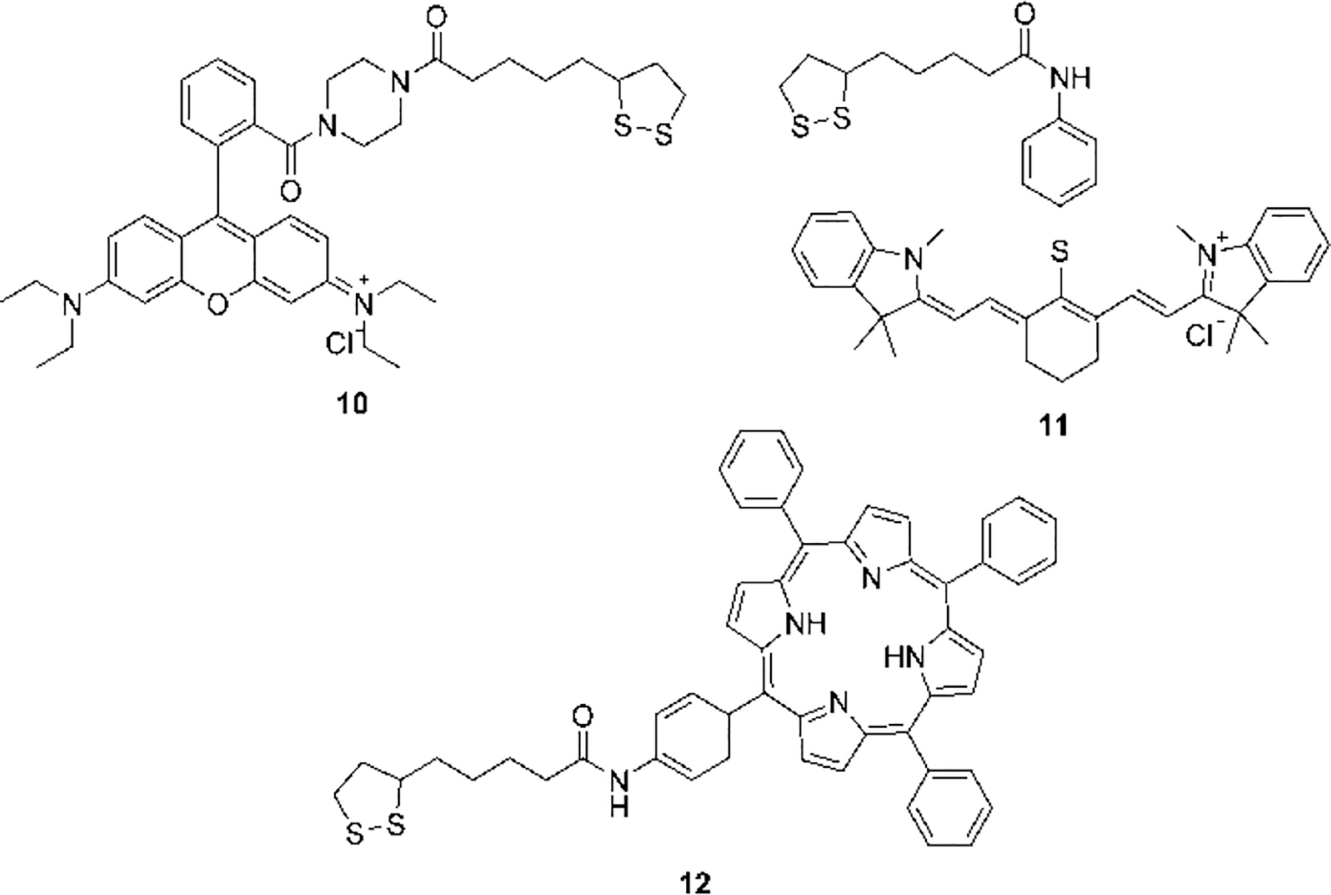
Fluorescent lipoic acid derivatives used for nanoparticle labeling.

Fluorescent labeling of the NPs was performed to directly study their biological localization. To avoid potential interference arising from autofluorescence of biological structures,^44^ fluorescence labels featuring long-wavelength absorption and emission were selected and conjugated with lipoic acid derivatives. We focused our attention to three molecules: rhodamine (**10**), cyanine dye IR-775 (**11**), and porphyrin (**12**) (Figure 5). These three dyes cover a large range of excitation and emission wavelengths in the visible and NIR regions.^45^ Indeed, the rhodamine derivative **10** emits at 593 nm (excitation 561 nm), the cyanine **11** at 816 nm (excitation at 775 nm), and the porphyrin **12** at 651 nm (excitation at 417 nm). Derivatives **10**–**12** were prepared as described in the Supporting Information and effectively copolymerized with compound **7**, resulting in the corresponding dye-doped nanoparticles. In each case, the emission of doped nanoparticles is similar to that of the corresponding monomer unit (Figures S4, S6, and S8).

### Thiol-Induced Degradation

As mentioned before, polydisulfide NPs are expected to undergo depolymerization in the presence of high concentrations of thiolates^22b^ (e.g., in glutathione and cysteine inside tumor cells). Therefore, we studied the effect of incubation with dithiothreitol and glutathione as reducing agents on size and NPs.

**Figure 6:**
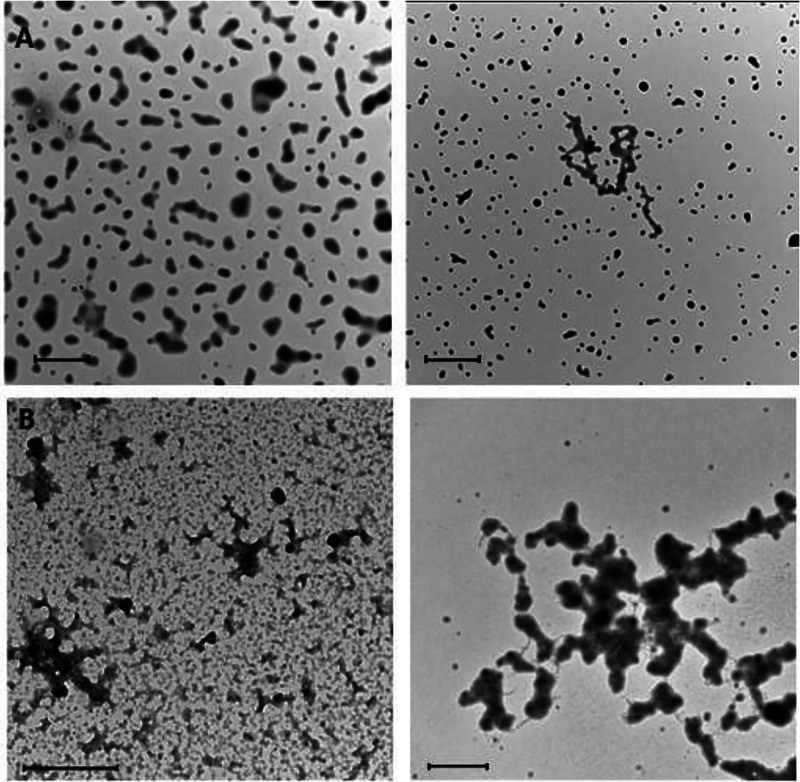
(A) TEM micrographs of F127@**7**-NPs doped with **10** (5% with respect to **7**, average size 120 nm, PDI: 0.088 measured by DLS, left) and of F127@**9**-NPs doped with **10** (5% with respect to **9**, average size 102 nm, PDI: 0.088 measured by DLS; right) incubated at 37 °C for 1 h in 10 mM glutathione. (B) TEM micrographs of F127@**7**-NPs doped with **10** (5% with respect to **7**, average size 110 nm, PDI: 0.046 measured by DLS) incubated at 37 °C for 24 h in 10 mM glutathione (left) and 10 mM DTT (right). Scale bars: 1 μm (A), 500 nm (B, left), and 2 μm (C, right).

Rhodamine-labeled F127@**7**-NPs and F127@9 were incubated with 10 mM dithiothreitol (DTT) or glutathione (GSH) at 37 °C for 1 and 24 h and their size and morphology were monitored via TEM and DLS. TEM analysis revealed fast degradation of the NPs, which lost their structure and formed small fragments or aggregates already after 1 h of incubation (Figure 6A). Interestingly, degradation of less cross-linked F127@**7** NPS was faster than that of F127@**9** NPs, suggesting that more extended cross-linking might provide some resistance to the loss of structure. No degradation was observed after 1 h incubation with 1 mM GSH at 37 °C (Figure S20) both with F127@**7** and F127@**9**. Prolonged incubation (24 h) at 10 mM thiols concentration resulted in extensive nanoparticles degradation (Figure 6B). In the case of NPs incubated with a lower (1 mM) GSH concentration, degradation was detected after 30 days of incubation.

DLS experiments, on the other hand, showed no apparent changes in the average diameter upon 24 h incubation.

The apparent discrepancy between the two experiments may be explained by the difference in the condition of analysis. The DLS measurements are performed in aqueous solutions, and the thiolate-induced depolymerization is expected to convert the cross-linked polymer chain into the lipoic monomers,^22^ which are still water-insoluble molecules. Hence, depolymerization may simply turn cross-linked polymeric nanoparticles into organic nanoparticles, with no apparent effect on the hydrodynamic size (Figure S19). This hypothesis is confirmed by the behavior observed with nanoparticles prepared in the absence of the thiol initiator, as previously discussed, which showed a relevant size increase only upon several days of incubation in water. On the other hand, TEM is performed after solvent evaporation and under high vacuum. In such conditions, molecular aggregates and monomers are expected to dissociate or, at least, lose their integrity as long as depolymerization proceeds and the cross-linked network degrades. Hence, degradation is positively observed with this technique.

We also preliminarily assessed the release of dye **10** from the nanoparticles in the presence of thiols. F127@**7** and F127@**9** NPs were incubated at 37 °C in the presence of 1 and 10 mM GSH for 1 h. Then, nanoparticles were isolated by centrifugation, resuspended, and the concentration of remaining dye measured. After incubation with 1 mM GSH, no dye leaching was detected. On the other hand, after incubation with 10 mM GSH, the dye loading in the nanoparticles decreased by 12% and 8%, respectively, for F127@**7** and F127@**9** NPs (Table S4).

### Freeze-Drying Stability

To confirm their potential as drug carriers, polymeric nanoparticles should demonstrate a high stability upon storage. Freeze-drying is a commonly used technique to store pharmaceutical preparations.^42^ Therefore, we tested the nanoparticles stability of rhodamine-labeled F127@**7**-NPs (average size 84 nm, PDI: 0.08 measured by DLS) toward lyophilization in the presence of different amounts of trehalose (5%, 10%, 25%, and 30%) as a lyoprotectant (Figure S22).^46^

Sugar molecules should interact with the solvent and PEG shell by hydrogen bonding, protecting the latter from the loss of adsorbed water molecules.^47^ Remarkably, the lack of the lyoprotectant resulted in a drastic increase in the nanoparticles’ dimension by 20-fold. On the other hand, the use of trehalose prevents particles aggregation. Reconstituted suspensions analyzed by DLS featured similar average diameter and slightly larger PDI than the as prepared one, indicating only minor NPs aggregation. These results confirmed the efficacy of the lyophilization technique for long-term preservation and storage of these polylipoic particles.

### Interaction with Serum/Plasma Proteins

The formation of a hard corona, a set of strongly bound biomolecules on the surface of NPs, is a possible source of interfering effects and adverse reactions in biological environments.

The protein corona has been indeed reported to affect cell interactions and the targeting selectivity of nanoparticles.^48^ In addition, some NP-bound proteins could favor inflammatory or procoagulant effects and the clearance by RES macrophages, especially the liver Kupfer cells.^49^ According to the most recent literature, polymer coating in many cases does not prevent protein binding, but controls the selective recruitment of a small pool of proteins that are, in turn, responsible for the nanoparticle’s properties.^50−53^

**Figure 7:**
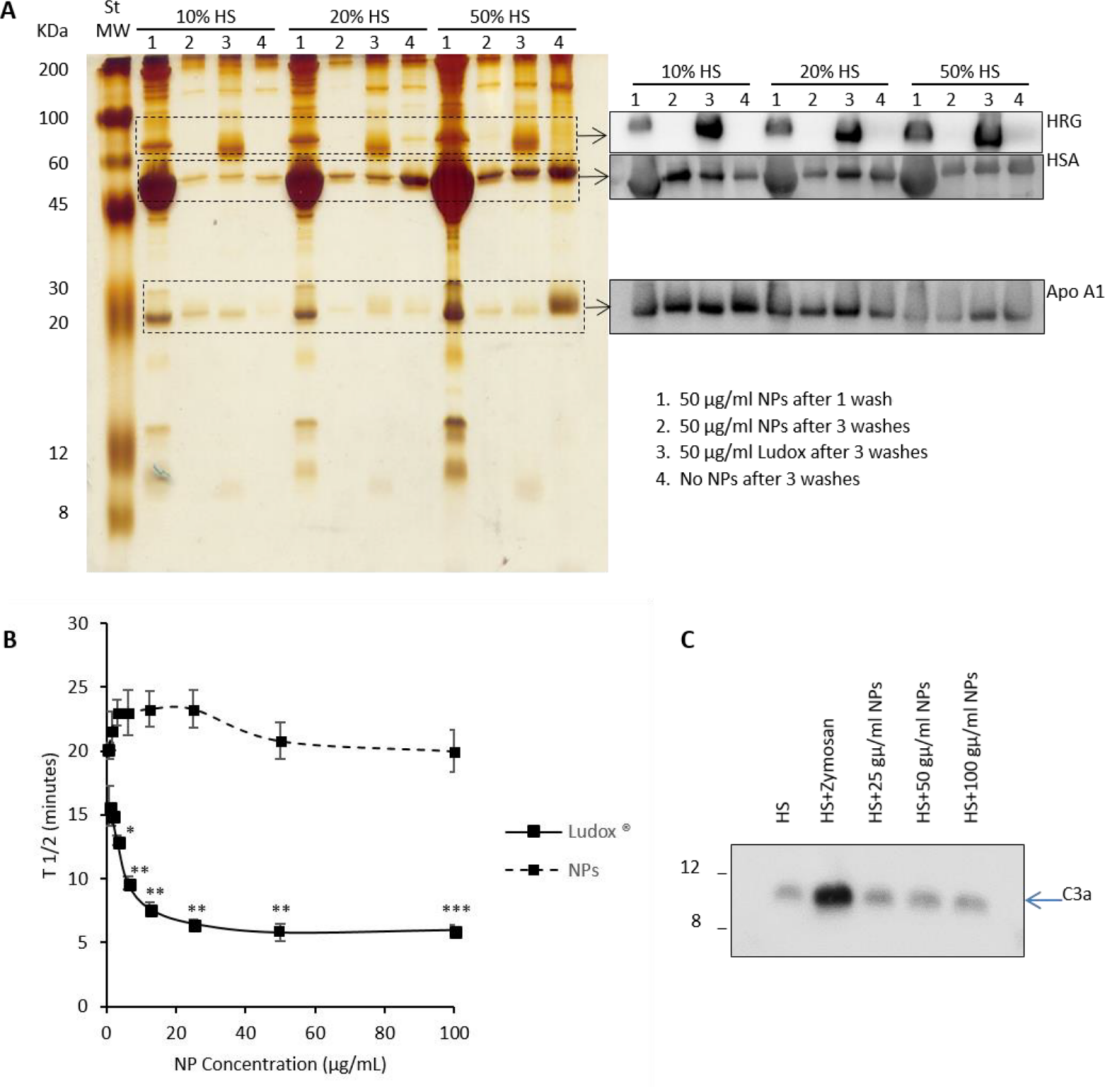
(A) silver staining (left panel) and Western blot analysis (right panel) of proteins associated to F127@**7**-NPs doped with **10** (5% with respect to **7**, average size 110 nm, PDI: 0.046 measured by DLS) or Ludox after 30 min incubation at 37 °C with different concentrations of HS (human serum); (B) the time corresponding to half maximal coagulation of HP deduced by kinetics curves in the presence of indicated NP and Ludox concentrations are shown; *p < 0.05; **p < 0.01; ***p < 0.001 with respect spontaneous coagulation; (C) C3a detection by Western blot in HS treated with Zymosan (positive control) or different NP concentrations.

The protein corona formed on our nanoparticles was investigated using SDS-PAGE analysis followed by sensitive silver staining. The experiment revealed that F127@**7**-NPs, preincubated with human serum (HS) up to quasi-physiological concentrations (50% v/v), were virtually free of bound serum proteins upon few washes in protein-free PBS (Figure 7A, left panel). The extent of protein association to poly(lipoic acid) nanoparticles after incubation with human plasma, human serum, and FCS was not dissimilar, or even reduced, compared to the one observed with 50:50 poly lactic-*co*-glycolic acid (PLGA) NPs (average size 95 nm, PDI 0.27 measured by DLS), functionalized with PEG_5000_, used as a regulatory-approved NP benchmark^57^ (Figures S23 and S24). On the contrary, amorphous Ludox-NPs, here used as a hard corona-endowed NP control, recruited and retained the major corona protein HRG even after washings, as previously demonstrated.^53^

Additional Western Blot (Wb) analysis, performed on typical corona-forming serum proteins albumin and Apo A1, as well as on HRG, also indicated a nonsignificant absorption of these proteins to F127@**7**-NPs (Figure 7A, right panel). Similar conclusions could be drawn when experiments were performed in human citrated plasma (HP) or in fetal calf serum (FCS; Figure S23A,B). Even in these two media, NPs did not show a strong protein binding propensity.

The binding to nanoparticles of selected plasma components may also trigger self-amplifying proteolytic cascades, primarily the coagulation process^54^ and the complement activation.^55^ These may be responsible for severe adverse reactions, like disseminated intravascular coagulation and inflammation and could also accelerate the clearance by RES macrophages.

However, consistently with the experiments showing a reduced protein recruitment, F127@**7**-NPs also showed a poor ability to trigger fibrin activation in human plasma and to form a clot via the FXII-dependent contact cascade, after reintroduction of physiological Ca^2+^. On the contrary, in the same conditions, the positive control Ludox-NPs was extremely effective (Figures 7B and S25).^56^ Moreover, the formation of the C3a anaphylotoxin, the active peptide released upon C3 cleavage and diagnostic of complement cascade activation, assayed by Wb after incubation of NPs with human serum was not increased compared to negative control (no agonist) by F127@**7**-NPs (Figure 7C).

We conclude that poly(lipoic acid)-based NPs do not form a stable protein corona and do not activate the contact coagulation and the complement cascades in human plasma/serum. As mentioned, several studies have recently demonstrated that densely PEGylated nanoparticles still retain the ability to recruit proteins from serum.^58^ The observation here reported that F127@**7**-NPs, which also feature a PEGylated surface, do not form a hard corona suggests that also the core material (not completely masked by the coating) might play a role in protein recruitment. The combined effect of the coating and the core material properties make these nanoparticles peculiarly inert toward the proteins present in human blood plasma.

### Compatibility of Poly(lipoic acid)-Based NPs with Blood Cells

**Figure 8:**
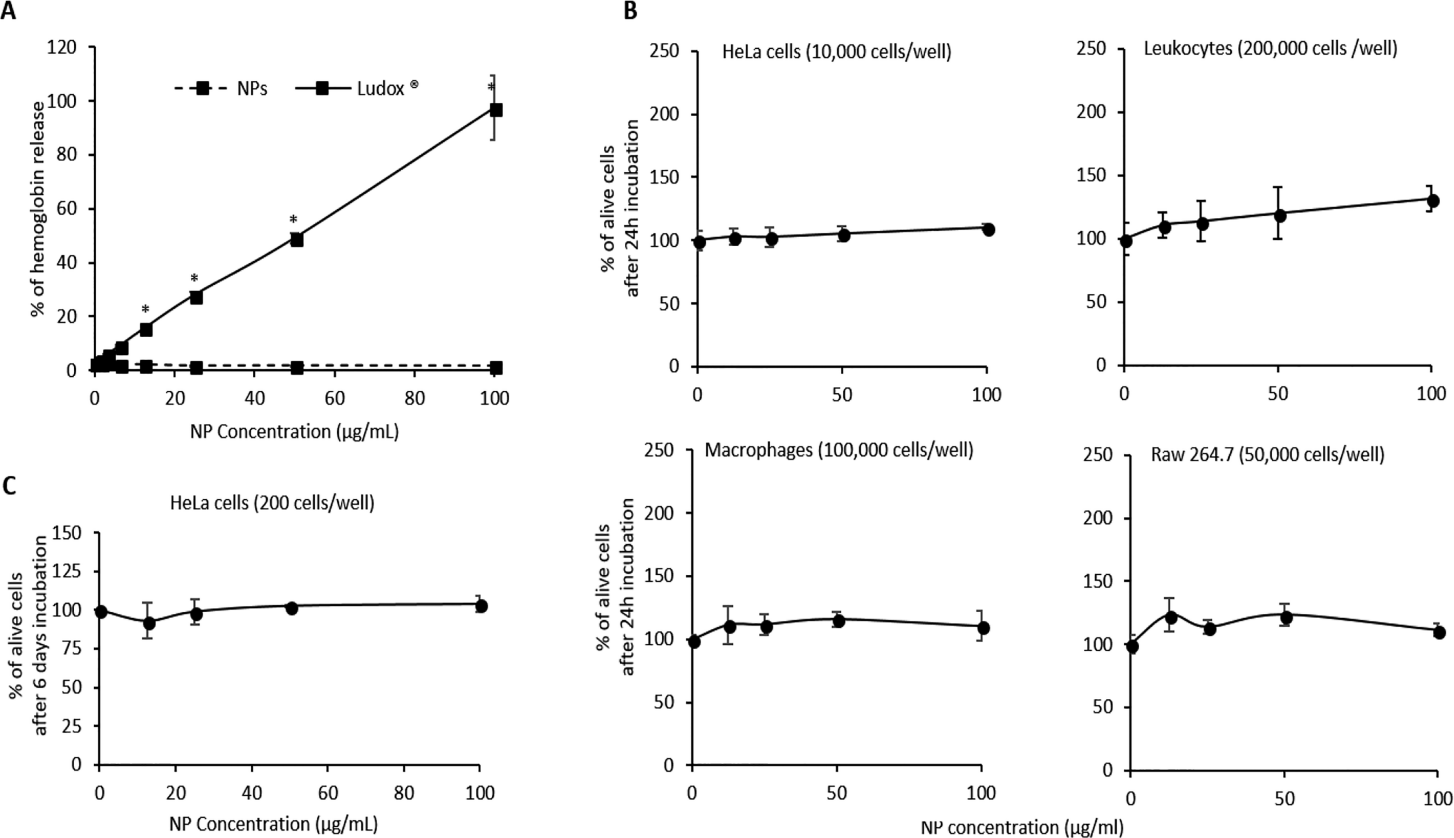
(A) Induction of hemolysis by different concentrations of F127@**7**-NPs doped with **10** (5% with respect to **7**, average size 110 nm, PDI: 0.046 measured by DLS) or Ludox after 2 h of incubation with fresh red blood cells; results were expressed as a percentage with respect to total hemolysis induced by water; *p < 0.05 with respect to the control sample. (B) Percentage of alive cells (with respect to not treated cells) after a 24 h treatment with different concentrations of NPs. (C) Percentage of alive cells (with respect to nontreated cells) after a 6 day treatment with different concentrations of NPs.

To further test in vitro the hemocompatibility of our NPs, we analyzed the possible induction of human erythrocytes lysis. No significant hemoglobin release was determined by F127@**7**-NPs, while in the same conditions Ludox-NPs were very effective and induced full erythrocyte disruption at the maximal dose tested (100 μg/mL; Figure 8A).

Subsequently, the presence of acute cytotoxic effects of F127@**7**-NPs after a 24 h incubation with pooled blood leukocytes, human and murine macrophages and the HeLa epithelial cell line, was tested by estimating their metabolic activity (MTT assay; Figure 8B). Again, and consistent with hemolytic assays, no effects were observed. Accordingly, the proliferation rate of HeLa cells, seeded at low density and allowed to grow in the presence of NPs for up to 6 days, was also not affected (Figure 8C).

We conclude that our poly(lipoic acid)-based NPs lack acute membrane-damaging and cytotoxic effects on red and white blood cells, but also on macrophages and epithelial cells. In addition, the proliferation rate of HeLa cells was perfectly normal after prolonged incubation with NPs, suggesting their biocompatibility even upon chronic cellular exposition.

### Interaction of NPs with Serum and Tissue In Vivo

Hemocompatibility and toxicity of our NPs were also studied in vivo in rat models. First, we analyzed the amount of serum neutrophil gelatinase-associated lipocalin (NGAL), a 25 kDa protein considered to be a sensitive biomarker for acute kidney injury and nonrenal organ damage. Tissues injured by ischemia or other toxic insults release NGAL as a protective agent against oxidative stress and as an activator of the regeneration processes.

**Figure 9:**
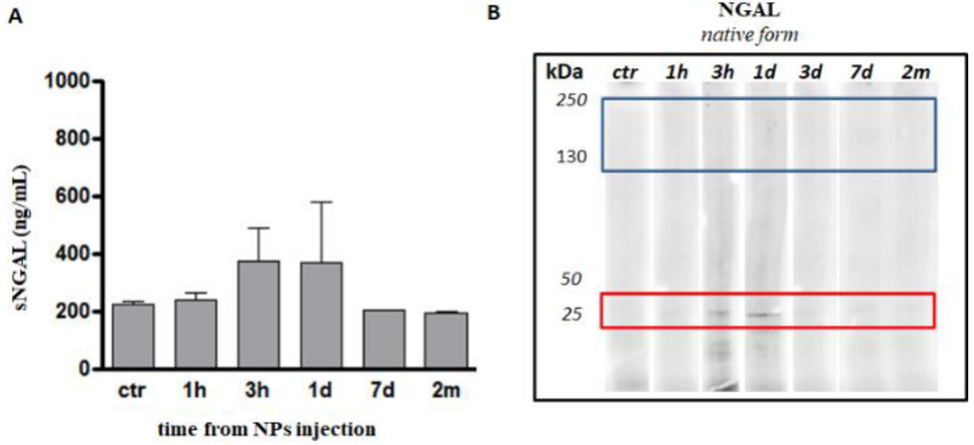
Evaluation of NPs toxicity in vivo. (A) sNGAL values on rat sera at different time points after F127@**7**-NPs tail vein injection. Note that sNGAL levels at 3 h until 1 day are significantly elevated compared to controls (375.3 ± 11.6 and 370.5 ± 209.5 ng/mL 3 h and 1 day, respectively). At 3/7 days to 2 months sNGAL levels returns to control values (201.6 ± 37.69 vs 224.5 ± 7.71 ng/mL, data for 7 days and basal rat level, respectively); (B) NGAL tissue-expression in the heart. Western blot in nonreducing conditions reveals an increase of 25 kDa bands, which correspond to monomers of NGAL protein at 3 h and 1 day from NPs injection. No NGAL protein bands have been noticed from 3 days up to 2 months after NPs injection.

As a consequence, high serum NGAL levels are diagnostic of chronic organ damage.^59^ Upon injection of 2 mg/rat F127@**7**-NPs, the basal rat serum NGAL levels (224.5 ± 7.71 ng/mL) slightly increased after 3 h (375.3 ± 11.6 ng/mL), to remain constant, albeit with relevant variability, up to 1 day (370.5 ± 209.5 ng/mL). This increase is smaller than those (up to 3-fold) observed with other factors inducing permanent damages.^59^ NGAL levels returned to normal basal values after 7 days (201.6 ± 37.69 ng/mL) and remained constant for at least 2 months (194.0 ± 4.0 ng/mL; Figure 9A).

To understand if the moderate and short reaction evidenced by the serum NGAL levels was related to any relevant tissue toxicity, we further evaluated the expression of NGAL protein in the cardiac tissue of the injected rats using Western Blot assays. Three isoforms of NGAL are usually isolated from tissues: the 25 kDa monomer (which is detected also in the serum), a 45 kDa disulfide-linked homodimer, and a 135–150 kDa heterodimer. The latter form is pathologically very relevant. Indeed, it consists of two components: a NGAL monomer covalently bound to a matrix metalloproteinase (in particular, MMP9).^59^ The formation of the dimer blocks the metalloproteinase activity, leading to collagen breakdown with negative heart remodeling. Western Blot analysis (Figure 9B) did not show any NGAL bands at 130–150 kDa, indicating the absence of a complex formation with MMP9 and, consequently, of a heart negative remodeling at any time from NPs injection (Figure 9B, blue square).

Moreover, in the heart of the injected rats, NGAL protein showed an increase of the 25 kDa form at 3 h and 1 day after NPs injection (Figure 9B, red square), in agreement with the observations performed on serum. This band quickly faded at longer times, to reach basal levels after only 3 days.

These results indicate that our poly(lipoic acid)-based NPs, not eliciting a significant and stable tissue toxicity over time, may be suitable as nanovectors also in heart targeting.

### Biodistribution Study In Vivo

**Figure 10:**
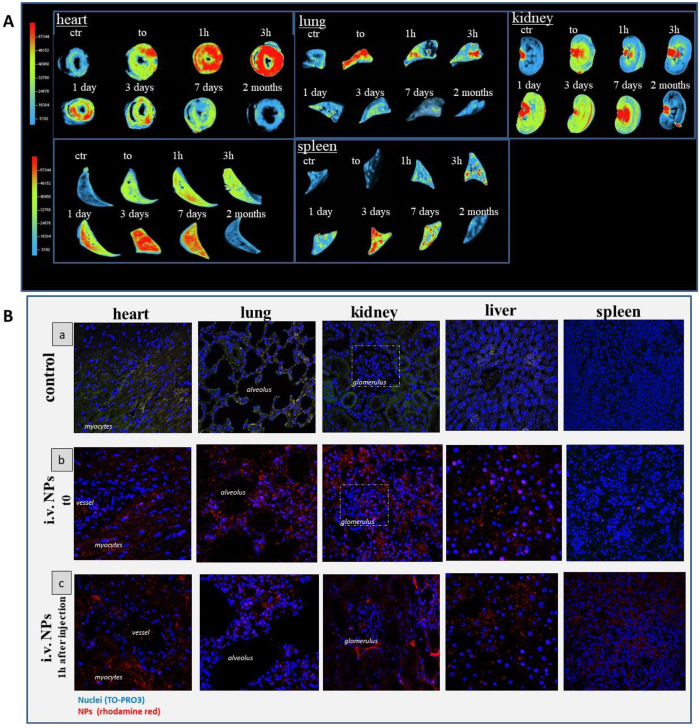
(A) Macroscopic organ biodistribution of NPs. Macroscopic epifluorescence images of NPs in different tissue sections at different time points after tail vein injection. The intensity of fluorescence of conjugated rhodamine red-NPs is represented, normalized, as pseudocolor scale bar that is consistent for all images. (B) Microscopic tissue NPs distribution: (a) Representative confocal laser microscopy images of control organs. The green/yellow color is due to the background of the tissue structures. In the heart image the green color, indicate mainly the myocytes. In the kidney image, the white dashed square indicates the glomerulus. In the liver image, the lobular liver structure can recognized. Nuclei are counterstain with TO-PRO3 in blue. Zoom from original magnification of 40×; (b) Laser confocal microscopy images depicting the tissue NPs detection at t0, immediately after i.v. injection. Note as NPs are more concentrated in heart, lung, liver, and kidney. In the kidney image, the white dashed square indicates the glomerulus. Nuclei are counterstain with TO-PRO3 in blue and conjugated rhodamine-red NPs are identified by red signal. Zoom from original magnification of 40×; (c) Representative laser confocal images 1 h after NPs injection, showing that nanoparticles (red signal) can reach the interstitium of the organs especially the heart. Moreover, 1 h after injection, the lung showed few NPs uptake compared to the other organs, which is in keeping with the macro-distribution analysis in (A). Nuclei are counterstain with TO-PRO3 (in blue) and conjugated rhodamine red-NPs were are identified in red. Zoom from original magnification of 40×.

We finally assessed the biodistribution of nanoparticles after injection by fluorescence imaging of sliced organs. This technique allows the visualization of the spatiotemporal distribution of NPs in different tissues. Specifically, the time-dependent biomolecular distribution imaging of rhodamine-labeled F127@**7**-NPs was obtained using Chroma Alliance 2.7 and its 3D software. Results, reported in Figure 10A, show the NPs distribution and clearance in the different organs at different times.

After injection, NPs rapidly spread in the rats being detected first in the lungs, then in the heart, and subsequently in the spleen and liver and, eventually, in the kidneys. Complete NPs clearance is reached after 7 days from NPs administration, showing organ epifluorescence signals similar to controls.

Remarkably, the heart seems to retain the greatest amount of NPs from 1 h up to 1 day after tail-vein injection, possibly as a result of the preferential cardiac metabolism for lipid substrates. Subsequent accumulation in spleen, liver, and kidneys is observed, likely due to the “filtering” ability of these organs. Lungs show fewer NP uptake compared to the other organs, being the short-term accumulation spike (at t_0_) likely due to mucociliary clearance.^60^ Short-term accumulation in the heart is not accompanied by negative effects, as confirmed by the previous NGAL experiments and visual inspection. Confocal microscopy images of rhodamine labeled F127@**7**-NPs in different tissues confirmed the signals observed in biodistribution analyses with nanoparticles reaching all the major organs but the spleen immediately after injection (t_0_) and being retained mostly by the heart, but also by liver and spleen, after 1 h. In addition, confocal experiments performed after 1 h also demonstrated the ability of NPs to reach the interstitium of the organs, especially the cardiac one (Figure 10B).

## Conclusions

We reported a novel, biocompatible and biodegradable polymeric nanosystem with properties remarkable for biomedical applications. The nanoprecipitation method combined with the RODEP reaction proved to be a very precise tool to control the nanoparticles assembly and polymeric core formation in aqueous media. At a difference from other poly(lipoic acid)-based nanoparticles, a one-pot preparation of loaded and PEG-coated nanoparticles is possible, with an evident advantage for large-scale production.

As known for the nanoprecipitation protocol, there are several parameters that influence the formation of the nanoparticles and their effect is often difficult to rationalize. Here, we found that besides the volume ratios of the solution mixed, the choice of stabilizing surfactant is apparently the crucial factor. Indeed, Pluronic F68 and F127, having very similar PEG to PPO ratios, gave comparable results, while P123 was unable to stabilize the nanoparticles. A minor role is also played by the chemical nature of the precursors, with larger and presumably more hydrophobic precursors forming slightly smaller nanoparticles.

The easy functionalization of lipoic acid with various fluorophores and their successful inclusion in the nanoparticles confirmed the synthetic versatility of this system, which opens the possibility of an easy doping with active species or drugs, as well as the preparation of multifunctional systems.

These nanoparticles were degradable in the presence of thiols, and this should entail fast payload release once taken up by cells. Also, their degradation should result in the release of lipoic acid, which may contribute to antioxidant cell pathways boosting the therapeutic effects.

As expected, the cell toxicity tests showed an excellent in vitro and in vivo biocompatibility. A remarkable and unexpected finding was that F127-coated NP did not form any hard protein corona, and, consistently, did not activate both the coagulation and the complement cascade. Such an effect is superior to what is currently observed with other PEGylated nanosystems and may give a fundamental contribution by prolongation of the in-blood nanoparticles circulation, resulting in more effective therapy, as well as in reducing unwanted immune response. Another striking feature was their early (1–3 h) accumulation in the heart in the preclinical rat model, which makes them a candidate as suitable vectors for cardiovascular applications.

All this information can be useful in the development of new patient-personalized drug delivery systems.

## Supporting Information Available

Synthesis and characterization of compounds **1**–**12**, general synthesis of polylipoic NP, chemical characterization, and in vitro and in vivo characterization of polylipoic NPs (PDF)
