# Supplementary material for: Poly(lipoic acid)-Based Nanoparticles as Self-Organized, Biocompatible, and Corona-Free Nanovectors
Source: Biomacromolecules. 2020 Dec 21;22(2):467–80. doi: 10.1021/acs.biomac.0c01321 (PMC8016167; doi:10.1021/acs.biomac.0c01321)
Supplement: Supplementary file 1 — bm0c01321_si_001.pdf [file bm0c01321_si_001.pdf]

Poly-lipoic acid-based nanoparticles as self-organized, biocompatible, and corona-free nanovectors

Jakub W. Trzciński,[a] Lucía Morillas-Becerril,[a] Sara Scarpa,[b,c] Marco Tannorella,[a] Francesco Muraca,[a] Federico Rastrelli,[a] Chiara Castellani,[d] Marny Fedrigo,[d] Annalisa Angelini,[d] Regina Tavano,[*,b,c] Emanuele Papini,[b,c] and Fabrizio Mancin[*,a]

[a] Dipartimento di Scienze Chimiche, Università di Padova, via Marzolo 1, Padova, I-35131, Italy. E-mail: fabrizio.mancin@unipd.it; Fax: +39 0498275239; Tel: +39 0498275666

[b] Dipartimento di Scienze Biomediche, Università di Padova, via U. Bassi 58/B1, Padova, I-35131, Italy. E-mail: regina.tavano@unipd.it; Fax: +39 0498276301; Tel: +39 0498276159

[c] Centre for Innovative Biotechnological Research-CRIBI, Università di Padova, via U. Bassi 58/B1, Padova, I-35131, Italy.

[d] Dipartimento di Scienze Cardio-Toraco-Vascolari e Sanità pubblica, Università di Padova, via Giustiniani 2, Padova, I-35128, Italy.

Table of contents

1. **General** 2
2. **Synthetic procedures** 2
3. **General synthesis of poly-lipoic nanoparticles** 12
4. **Chemical characterization of nanoparticles** 19
5. **In vitro characterization of nanoparticles** 24
6. **In vivo characterization of nanoparticles** 29
7. **References** 31

1. General

Chemical reagents were purchased from Aldrich at highest commercial quality and used without further purification. Water was purified using a Milli-Q® and water purification system. Reactions were monitored by TLC developed on 0.25 mm Merck silica gel plates (60 F254). Solvents were of analytical reagent grade, laboratory reagent grade or HPLC grade.

NMR spectra in the solution state were recorded on a AVIII 500 spectrometer (500 MHz for 1H frequency) or on a Bruker AC-300 (300 MHz for 1H frequency). UV-Vis absorption spectra were measured in methanol on a Varian Cary 50 spectrophotometer with 1 cm path length quartz cuvettes. Fluorescence spectra were measured in water or methanol on a Varian Cary Eclipse fluorescence spectrophotometer. Both the spectrophotometers were equipped with thermostatted cell holders. ESI-MS were recorded on Agilent Technologies 1100 Series system equipped with a binary pump (G1312A) and MSD SL Trap mass spectrometer (G2445D SL).

2. Synthetic procedures

1. Synthesis of 5-(1,2-dithiolan-3-yl)-N-propylpentanamide (1)

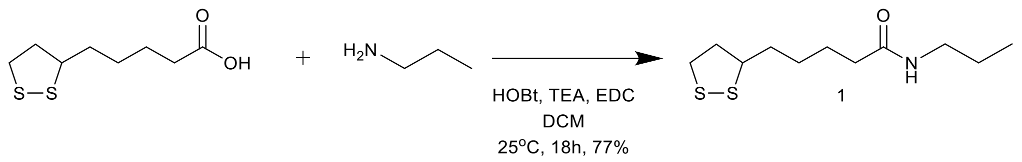

Lipoic acid (1 g, 4.85 mM), propylamine (0.34 mL, 5.81 mM), HOBt (0.78 g, 5.81 mM) and triethylamine (0.8 mL, 5.81 mM) were dissolved in 20 mL of anhydrous dichloromethane (DCM) under N_2 atmosphere and stirred at 0°C for 15min. Subsequently, EDC (1.11 g, 5.81 mM) was added and stirred overnight at 25°C in the dark. The resulting mixture was concentrated under reduced pressure and purified by column chromatography (silica gel, DCM:EtOAc 9:1 as eluent) yielding the product as yellow solid (0.92 g, 77%). ^{1}H NMR (300 MHz, $CDCl_3$) δ: 0.91 (3H, J = 7.4 Hz, t), 1.47 (2H, m), 1.50 (2H, J = 7.4 Hz, qn), 1.67 (4H, m), 1.90 (1H, m), 2.18 (2H, J = 7.4 Hz, t), 2.45 (1H, m), 3.14 (4H, m), 3.56 (1H, m), 5.59 (1H, bs).

2. Synthesis of N,N'-(ethane-1,2-diyl)bis(5-(1,2-dithiolan-3-yl)pentanamide) (2)

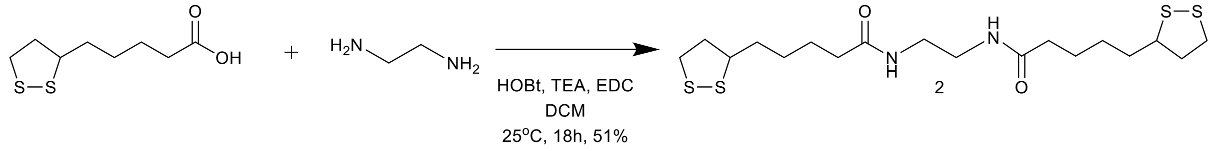

Lipoic acid (1 g, 4.84 mM), triethyleneamine (3.437 mL, 24.20 mM) and HOBt (1.144 g, 8.47 mM) were dissolved in 25 mL of anhydrous dichloromethane (DCM) under N_2 atmosphere and stirred at

0°C for 15 min. Subsequently, EDC (1.623 g, 8.47 mM) was added and stirred for 15 min. Ethylenediamine (145 mg, 4.42 mM) was added and the solution was maintained overnight at 25°C. The reaction mixture was extracted with 2x150 mL of 0,5 M citric acid, 2x150 mL of H_2O, 2x150 mL of 5% $NaHCO_3$, 1x150 mL of H_2O. The organic solution was dried over $MgSO_4$, and the solvent was removed in vacuo, yielding the product as a white solid (0.58 g, 51%). ^{1}H-NMR (300 MHz, $CDCl_3$) δ: 1.47 (4H, m), 1.66 (8H, m), 1.91-2.43 (4H, m), 2.22 (4H, J = 7.4 Hz, t), 3.12 (4H, m), 3.38 (4H, s), 3.55 (2H, m), 6.55 (2H, bs).

3. Synthesis of N,N'-(octane-1,8-diyl)bis(5-(1,2-dithiolan-3-yl)pentanamide) (3)

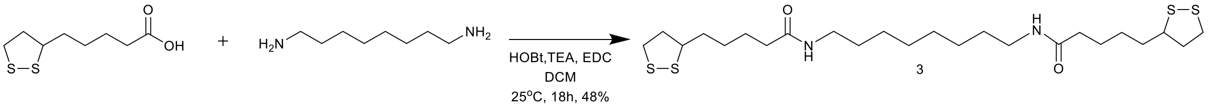

Lipoic acid (1 g, 4.84 mM), triethyleneamine (3.437 mL, 24.20 mM) and HOBt (1.144 g, 8.47 mM) were dissolved in 25 mL of anhydrous dichloromethane (DCM) under N_2 atmosphere and stirred at 0°C for 15 min. Subsequently, EDC (1.623 g, 8.47 mM) was added and stirred for 15 min. Octyldiamine (0.35 g, 2.42 mM) was added and the solution was maintained overnight at 25°C. The reaction mixture was exctracted with 2x150 mL of 0,5M citric acid, 2x150 mL of H_2O, 2x150 mL of 5% $NaHCO_3$, 1x150 mL of H_2O. The organic phase was dried over $MgSO_4$, and the solvent was removed in vacuo, yielding the product as a white solid (0.657 g, 48%). ^{1}H-NMR (300 MHz, $CDCl_3$) δ: 1.30 (8H, m), 1.47 (8H, m), 1.67 (8H, m), 1.90 (2H, m), 2.18 (4H, J = 7.4 Hz, t), 2.45 (2H, m), 3.14-3.26 (8H, m), 3.58 (2H, m), 6.50 (2H, bs).

4. Synthesis of N,N'-((ethane-1,2-diylbis(oxy))bis(ethane-2,1-diyl))bis(5-(1,2-dithiolan-3 -yl)pentanamide) (4)

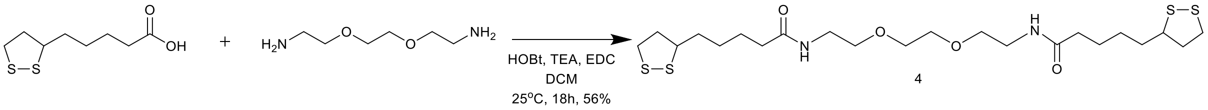

Lipoic acid (1 g, 4.84 mM), triethyleneamine (3.437 mL, 24.20 mM) and HOBt (1.144 g, 8.47 mM) were dissolved in 25 mL of anhydrous dichloromethane (DCM) under N_2 atmosphere and stirred at 0°C for 15 min. Subsequently, EDC (1.623 g, 8,47 mM) was added and stirred for 15 min. 2-[2-(2-aminoethoxy)ethoxy]ethaneamine (0.36 g, 2.42 mM) was added and the solution was maintained overnight at 25°C. The reaction was extracted with 2x150 mL of 0,5M citric acid, 2x150 mL of H_2O, 2x150 mL of 5% $NaHCO_3$, 1x150 mL of H_2O. The organic phase was dried over $MgSO_4$, and the solvent was removed in vacuo, yielding the product as white solid (0.76 g, 56%). ^{1}H-NMR (300 MHz, $CDCl_3$) δ: 2.47 (4H, m), 2.66 (8H, m), 2.91-3.45 (4H, m), 3.21 (4H, J = 7.4 Hz, t), 4.12 (4H, m), 4.32-4.61 (12H, m), 5.35 (2H, bs).

5. Synthesis of propyl 5-(1,2-dithiolan-3-yl)pentanoate (5)[1]

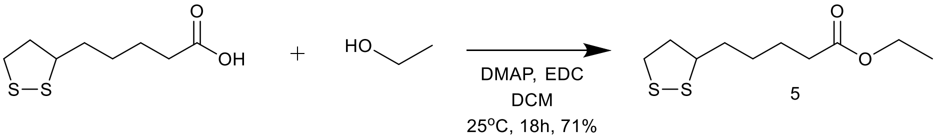

Lipoic acid (1 g, 4.85 mM), ethanol (0.34 mL, 5.81 mM) and DMAP (0.71 g, 5.81 mM) were dissolved in 20 mL of anhydrous dichloromethane (DCM) under N_2 atmosphere and stirred at 0°C for 15min. Subsequently, EDC (2.11 g, 5.81 mM) was added and stirred overnight at 25°C in the dark. The resulting mixture was concentrated under reduced pressure and purified by column chromatography (silica gel, DCM as eluent) yielding the product as yellow oil (0.81 g, 71%). ^{1}H NMR (300 MHz, $CDCl_3$) δ: 1.20 (3H, J = 7.1 Hz, t), 1.42 (2H, m), 1.62 (4H, m), 1.86 (1H, m), 2.26 (2H, J = 7.4 Hz, t), 2.41 (1H, m), 3.10 (2H, m), 3.52 (1H, m), 4.07 (2H, J = 7.1 Hz, q). Characterization data are consistent with literature.[1]

6. Synthesis of ethane-1,2-diyl bis(5-(1,2-dithiolan-3-yl)pentanoate) (6)[1]

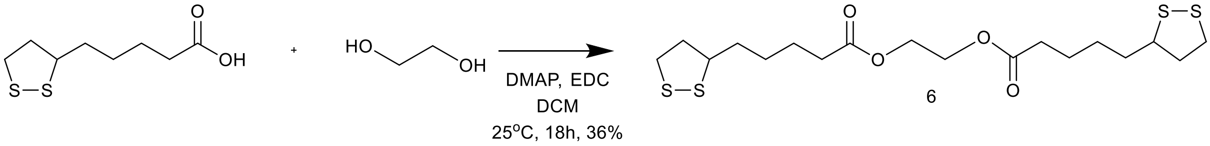

Lipoic acid (2.48 g, 12 mM), ethylene glycol (0.37 g, 6 mM) and DMAP (1.47 g, 12 mM) were dissolved in 20 mL of anhydrous dichloromethane (DCM) under N_2 atmosphere and stirred at 0°C for 15min. Subsequently, EDC (2.3 g, 12 mM) was added and stirred overnight at 25°C in the dark. The resulting mixture was filtered, concentrated under reduced pressure and purified by column chromatography (silica gel, $CHCl_3$:MeOH 9:1 as eluent) yielding the product as yellow oil (1 g, 36%). ^{1}H-NMR (300 MHz, $CDCl_3$) δ: 1.48 (4H, m), 1.67 (8H, m), 1.89 (2H, J = 12.4, 6.4 Hz, dq), 2.46 (2H, J = 12.4, 6.4 Hz, dq), 2.35 (4H, J = 7.4 Hz, t), 3.13 (4H, m), 3.56 (2H, m), 4.27(4H, s). Carachterization data are consistent with literature.[1]

7. Synthesis of octane-1,8-diyl bis(5-(1,2-dithiolan-3-yl)pentanoate) (7)[1]

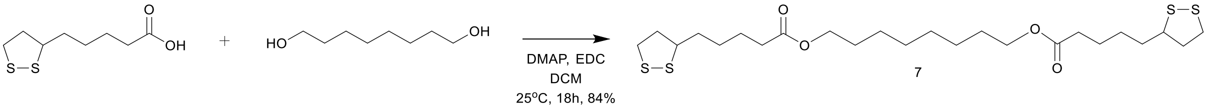

Lipoic acid (2.48 g, 12 mM), 1,8-octanediol (0.87 g, 6 mM) and DMAP (1.47 g, 12 mM) were dissolved in 20 mL of anhydrous dichloromethane (DCM) under N_2 atmosphere and stirred at 0°C for 15min. Subsequently, EDC (2.3 g, 12 mM) was added and stirred overnight at 25°C in the dark. The resulting mixture was filtered, concentrated under reduced pressure and purified by column chromatography (silica gel, DCM as eluent) yielding the product as yellow oil (2.7 g, 84%). ^{1}H-NMR, (500 MHz, $CDCl_3$) δ: 1.32 (8H, s), 1.46 (4H, m), 1.67 (12H, m), 1.91 (2H, J = 12.4, 6.4 Hz, dq), 2.31

(4H, J = 7.4 Hz, t), 2.46 (2H, J = 12.4, 6.4 Hz, dq), 3.15 (2H, m), 3.57 (2H, m), 4.06 (4H, J = 6.7 Hz, t). ^{13}C-NMR, (126 MHz, $CDCl_3$) δ: 24.74, 25.87, 28.62, 28.79, 29.14, 34.13, 34.63, 38.50, 40.23, 56.37, 64.46, 173.61. ESI-MS: 545.1 m/z ($M+Na^+$), 561.0 m/z ($M+K^+$). Characterization data are consistent with literature.[1]

8. Synthesis of (ethane-1,2-diylbis(oxy))bis(ethane-2,1-diyl) bis(5-(1,2-dithiolan-3-yl)pentanoate) (8) [1]

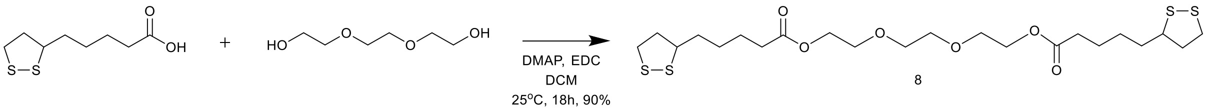

Lipoic acid (2.48 g, 12 mM), triethylene glycol (0.90 g, 6 mM) and DMAP (1.47 g, 12 mM) were dissolved in 20 mL of anhydrous dichloromethane (DCM) under N_2 atmosphere and stirred at 0°C for 15min. Subsequently, EDC (2.3 g, 12 mM) was added and stirred overnight at 25°C in the dark. The resulting mixture was filtered, concentrated under reduced pressure and purified by column chromatography (silica gel, $CHCl_3$:MeOH 10:0,5 as eluent) yielding the product as yellow oil (2.83 g, 90%). ^{1}H-NMR (300 MHz, $CDCl_3$) δ: 1.44 (4H, m), 1.66 (8H, m), 1.88 (2H, J = 12.5, 6.5 Hz, dq), 2.33 (4H, J = 7.4 Hz, t), 2.44 (2H, J = 12.5, 6.5 Hz, dq), 3.12 (4H, m), 3.54 (2H, m), 3.63 (4H, s), 3.67 (4H, m), 4.20 (4H, m). Characterization data are consistent with literature.[1]

9. Synthesis of propane-1,2,3-triyl tris(5-(1,2-dithiolan-3-yl)pentanoate) (9) [1]

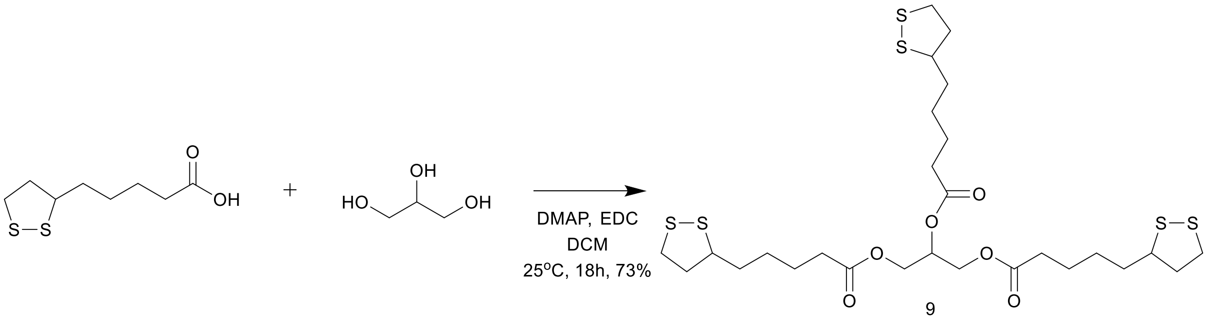

Lipoic acid (3 g, 14.5 mM), glycerol (0.45 g, 4.85 mM) and DMAP (1.78 g, 14.54 mM) were dissolved in 25 mL of anhydrous dichloromethane (DCM) under N_2 atmosphere and stirred at 0°C for 15min. Subsequently, EDC (2.8 g, 14.54 mM) was added and stirred overnight at 25°C in the dark. The resulting mixture was filtered, concentrated under reduced pressure and purified by column chromatography (silica gel, $CHCl_3$:EtOAc = 6:1 as eluent) yielding the product as a yellow oil (2.3 g, 73%). ^{1}H-NMR, (500 MHz, $CDCl_3$) δ: 1.38–1.56 (6H, m), 1.58–1.79 (12H, m), 1.91 (3H, J = 12.7, 6.9 Hz, dq), 2.34 (6H, m), 2.47 (3H, m), 3.06–3.24 (6H, m), 3.57 (1H, 8.7, 6.3 Hz, dq), 4.14 (2H, m), 4.31 (2H, J = 12.0, 4.2 Hz, dd), 5.26 (1H, m). ^{13}C-NMR, (126 MHz, $CDCl_3$) δ: 24,57, 33.80, 33.96, 34.60, 38.52, 40.26, 56.34, 62.17, 69.01, 172.93. ESI-MS: 679 m/z ($M+Na^+$), 695 m/z ($M+K^+$). Characterization data are consistent with literature.[1]

10.Synthesis of N-(9-(2-(4-(5-(1,2-dithiolan-3-yl)pentanoyl)piperazine-1-carbonyl)phenyl)-6-(diethylamino)-3H-xanthen-3-ylidene)-N-ethylethanaminium (10)[2]

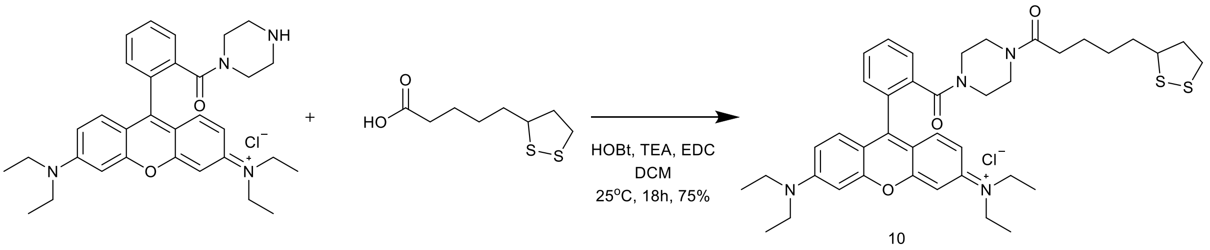

Lipoic acid (0.009 g, 0.0438 mM), triethyleneamine (0.006 mL, 0.0438 mM) and HOBT (0.006 g, 0.0444 mM) were dissolved in 10 mL of anhydrous dimethylformamide (DMF) under N_2 atmosphere and stirred at 0°C for 15min. Subsequently, EDC (0.084 g, 0.0438 mM) was added and stirred for 15 min. The rhodamine B piperazine amide (0.02 g, 0.0365 mM), prepared as reported,[2] was added and the solution was maintained overnight at 25°C. The mixture was concentrated under reduced pressure and purified by column chromatography (silica gel, DCM:MeOH = 9:1 as eluent yielding the product as a yellow oil (0.018 g, 75%). ^{1}H-NMR, (500 MHz, $CDCl_3$) δ: 1.32 (12H, (4H, J = 7.2 Hz, t), 1.37–1.53 (2H, m), 1.54–1.76 (4H, m), 1,83–1,97 (1H, m), 2.39 (2H, J = 7.4 Hz, t), 2.41–2.53 (1H, m), 3.05–3.21 (2H, m), 3.24–3.72 (16H, m), 6.62-7.76 (10H, m). ^{13}C-NMR, (126 MHz, $CDCl_3$) δ: 12.68, 23.41, 24.53, 24.83, 28.70, 28.89, 29.69, 32.95, 33.68, 34.60, 34.67, 38.49, 40.21, 40.97, 41.81, 42.27, 44.92, 46.15, 47.19, 48.14, 56.34, 56.47, 95.89, 96.73, 113.95, 114.96, 127.63, 130.32, 132.54, 155.72, 156.26, 157.73, 167.83, 176.82. UV-VIS: λ_{max} = 561 nm in MeOH. Fluorescence: λ_{em} = 593 nm in MeOH.

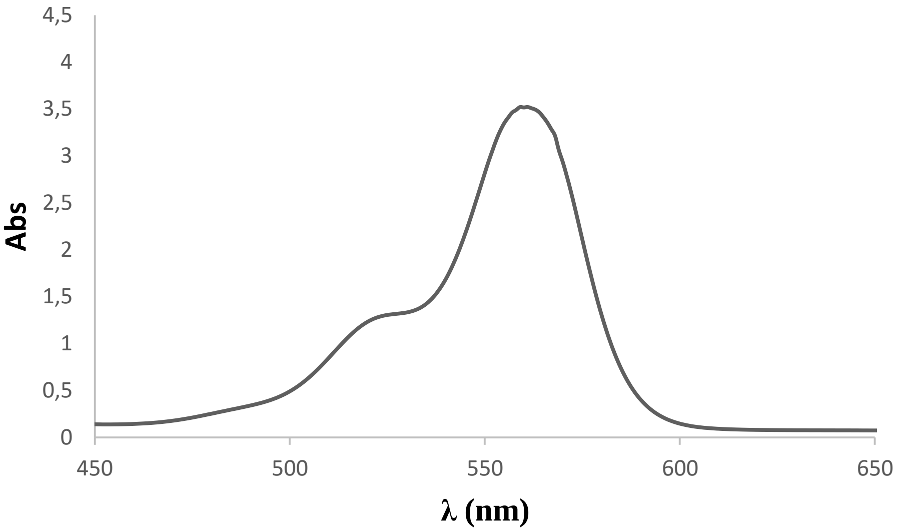

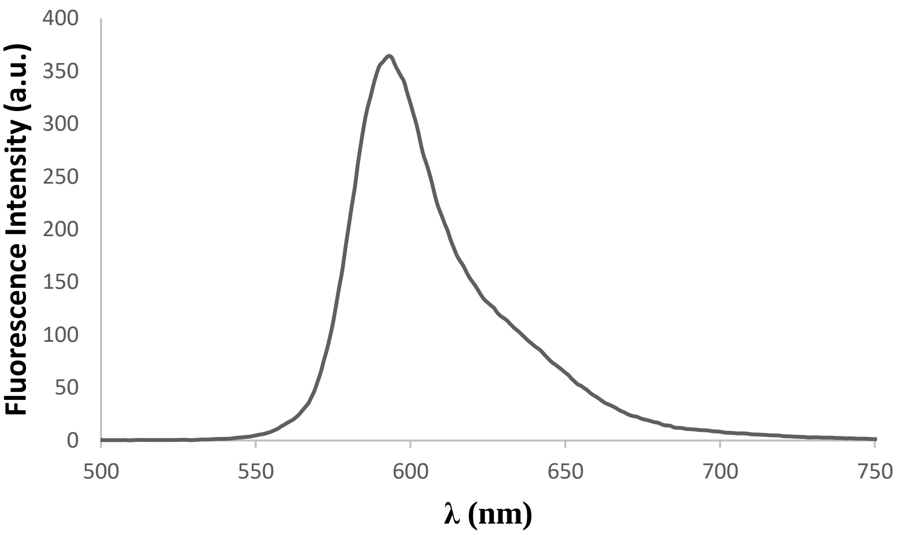

Figure S1: UV-VIS spectrum (top) and fluorescence emission spectrum (bottom) of compound **10** in MeOH**.**

11. Synthesis of 2-((E)-2-((E)-2-((4-(5-(1,2-dithiolan-3-yl)pentanamido)phenyl)thio)-3-(2-((Z)-1,3,3-trimethylindolin-2-ylidene)ethylidene)cyclohex-1-en-1-yl)vinyl)-1,3,3-trimethyl-3H-indol-1-ium chloride (11) [3]

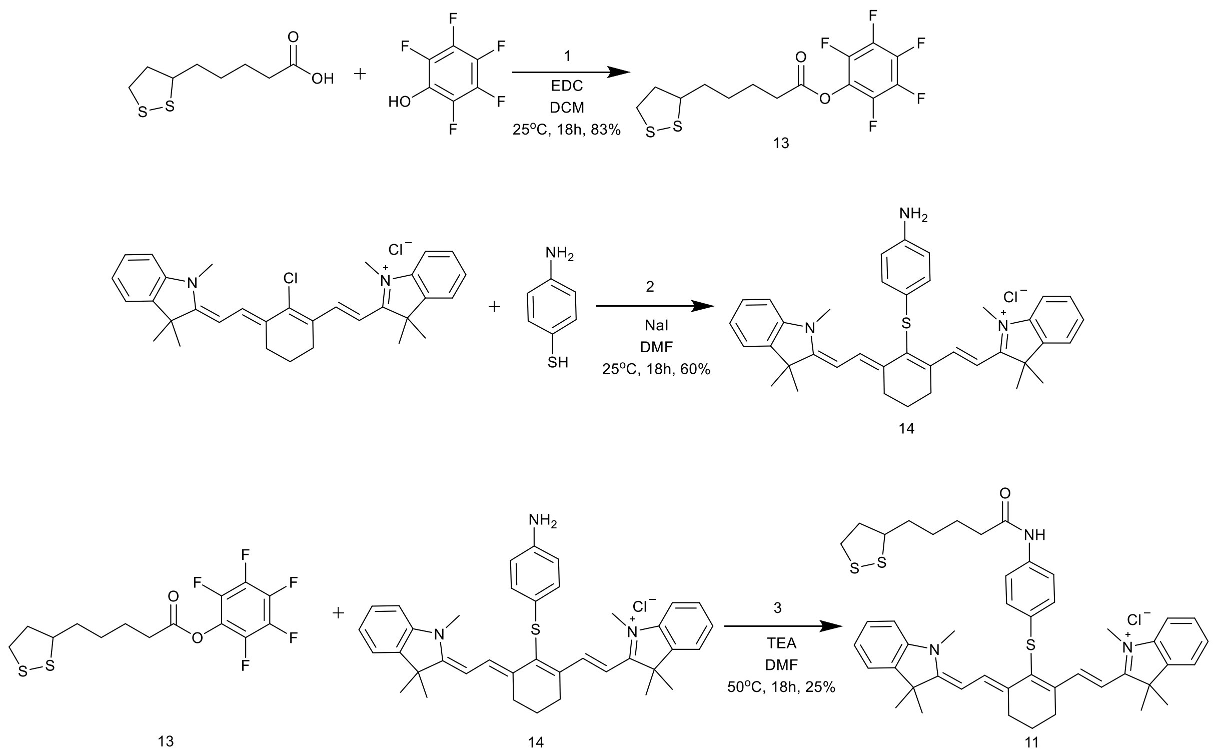

Synthesis of **11** has been divided in three steps:

Synthesis of perfluorophenyl 5-(1,2-dithiolan-3-yl)pentanoate (13). Lipoic acid (2 g, 9.7 mM), pentafluorophenol (2.1 g, 11.6 mM) were dissolved in 20 mL of anhydrous dichloromethane (DCM) under N_2 atmosphere and stirred at 0°C for 15min. Subsequently, EDC (2.2 g, 11.6 mM) was added and stirred for 15 min and the solution was maintained overnight at 25°C in the dark. The mixture was concentrated under reduced pressure and purified by column chromatography (silica gel, DCM:Petroleum Ether = 1:1 as eluent) yielding the product as a yellow oil (3 g, 83%). ^{1}H NMR (300 MHz, MeOD) δ: 1.36–1.76 (6H, m), 1.77–1.92 (2H, m), 2.39 (2H, m), 2.65 (2H, J = 7.4 Hz, t), 2.93–3.14 (2H, m), 3.51 (1H, m). ESI-MS: m/z: 373 (M+H^{+}).

Synthesis of 2-((E)-2-((E)-2-((4-aminophenyl)thio)-3-(2-((Z)-1,3,3-trimethylindolin-2-ylidene)ethylidene)cyclohex-1-en-1-yl)vinyl)-1,3,3-trimethyl-3H-indol-1-ium chloride (14). 4-aminothiophenol (0.24 g, 1.92 mM), IR-775 (0.1 g, 0.192 mM) were dissolved in 10 mL of anhydrous DMF under N_2 atmosphere and stirred 15min. Subsequently, NaI (0.054 g, 0.38 mM) was added and the solution was maintained overnight at 25°C. The mixture was concentrated under reduced pressure and purified by column chromatography (silica gel, DCM:MeOH = 9.8:2 as eluent), yielding the product as a dark-green powder (0.18 g, 60%). ^{1}H-NMR (500 MHz, DMSO) δ: 1.54 (12H, s), 1.88 (2H, m), 2.71 (2H, J = 6.5 Hz, t), 3.64 (6H, s), 5.18 (2H, bs), 6.27 (2H, J = 14,2 Hz, d), 6.51 (2H, J =

8.5 Hz, d), 6.98 (2H, J = 8.8 Hz d), 7.32 (2H, m), 7.41 (2H, m), 7.55 (2H, J = 8.1 Hz, d), 8.71 (2H, J = 14,2 Hz, d). ESI-MS: m/z: 572 (M^+).

Synthesis of 2-((E)-2-((E)-2-((4-(5-(1,2-dithiolan-3-yl)pentanamido)phenyl)thio)-3-(2-((Z)-1,3,3-trimethylindolin-2-ylidene)ethylidene)cyclohex-1-en-1-yl)vinyl)-1,3,3-trimethyl-3H-indol-1-ium chloride (11). Compound **13** (0.1 g, 0.272 mM), compound **14** (0.026 g, 0.045 mM) and Et_3N (0.031 mL, 0.226 mM) were dissolved in 10 mL of anhydrous DMF under N_2 atmosphere and stirred overnight at 50°C. The mixture was concentrated under reduced pressure and purified by column chromatography (silica gel, DCM:MeOH = 95:5 as eluent), yielding the product as a dark-green powder (0.013 g, 25%). ^{1}H NMR (500 MHz, DMSO) δ: 1.30 (6H, m), 1.46 (12H, s), 1.55 (2H, m). 1.81 (2H, m), 2.24 (2H, J = 7.5 Hz, t), 2.36 (2H, m), 2.75 (2H, J = 7.0 Hz, t), 3.09 (2H, m), 3.64 (6H, s), 6.30 (2H, J = 13.9 Hz, d), 7.19 (2H, J = 7.7 Hz, d), 7.40 (2H, J = 8.2 Hz, d), 7.72 (2H, m), 8.62 (2H, J = 13.9 Hz, d). ^{13}C NMR (126 MHz, DMSO) δ**:** 11.58, 20.93, 24.58-25.37 26.28, 27.78, 28.70, 34.48, 38.45, 45.46, 49.26, 56.52, 102.19, 111.90, 120.66, 122.88, 125.42, 126.87, 128.92, 133.63, 140.69-141.71, 142.67, 145.53, 172.56. ESI-MS: m/z: 760 (M^+). UV-Vis: λ_{max} = 782 nm in MeOH. Fluorescence: λ_{em} = 816nm in MeOH.

Figure S2: UV-VIS spectrum (top) and fluorescence emission spectrum (bottom) of compound **11** in MeOH**.**

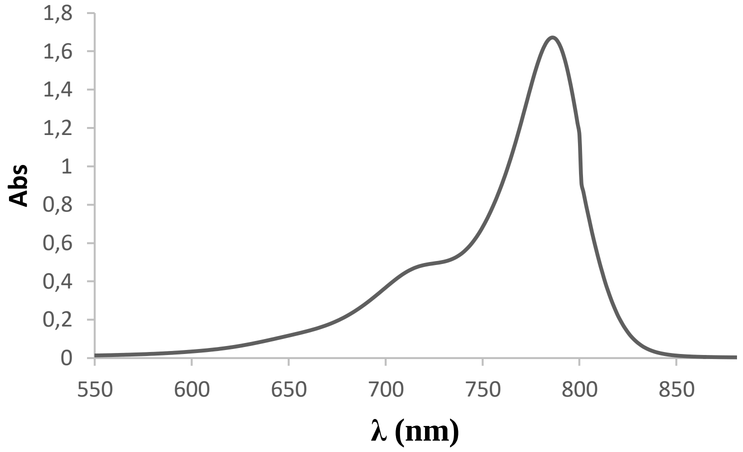

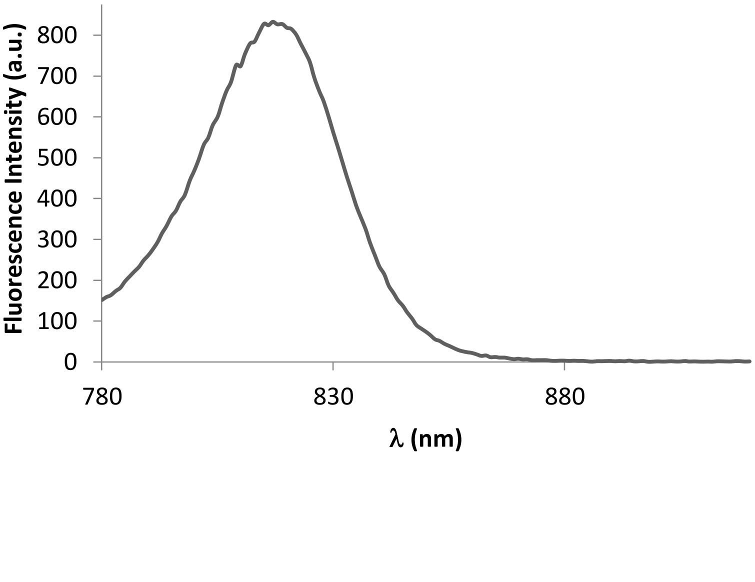

12. Synthesis of 5-(1,2-dithiolan-3-yl)-N-(4-(10,15,20-triphenylporphyrin-5-yl)phenyl) pentanamide (12) [4]

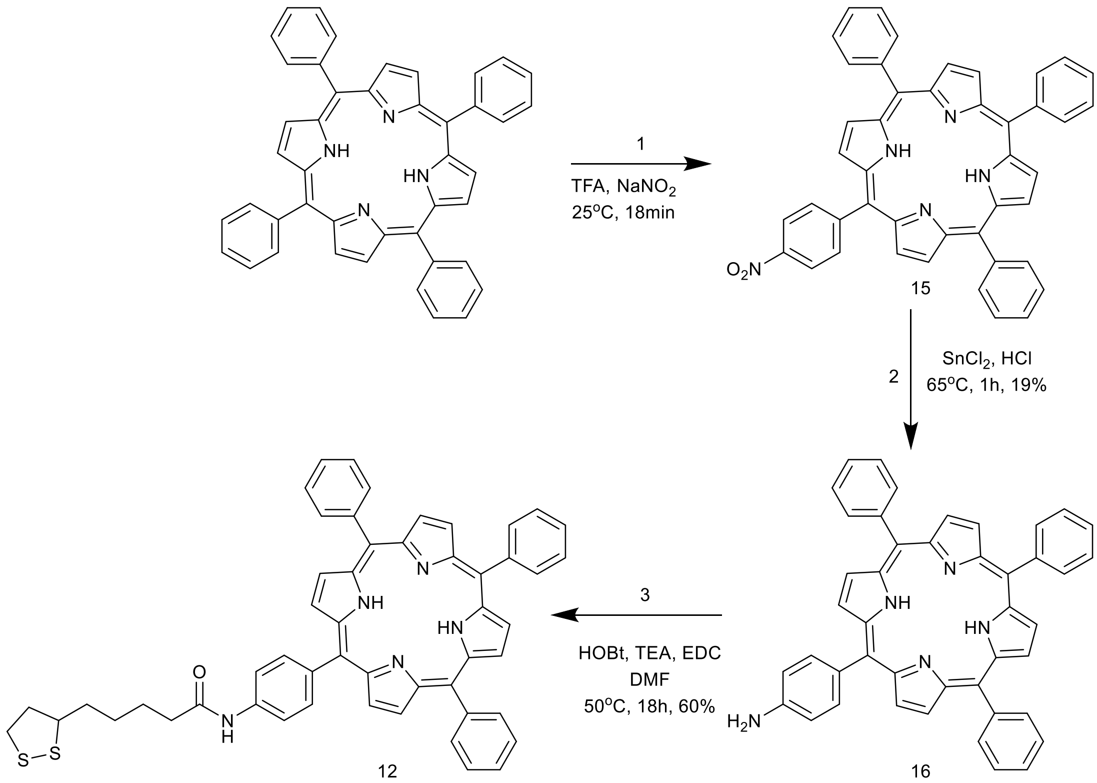

Synthesis of **12** has been divided in three steps:

Synthesis of 5-(4-nitrophenyl)-10,15,20-triphenylporphyrin (15). Tetraphenylporphyrin (0.5 g, 0.814 mM), was dissolved in 50 mL of trifluoroacetic acid (TFA) under N_2 atmosphere and stirred 15min. Subsequently. $NaNO_2$ (0.1 g, 0.37 mM) was added and the solution was stirred for 3 minutes at 25°C. The reaction was diluted by 500 mL of H_2O and the product was extracted 4 times by DCM. The organic fractions were collected. washed by saturate solution of $NaHCO_3$ followed by 4 washings with H_2O. The organic fractions were collected, dried over $MgSO_4$ and the solvent was removed in vacuo yielding the product as deep-green solid. The product was used for further reaction without purification.

Synthesis of 4-(10,15,20-triphenylporphyrin-5-yl)aniline (16). The crude compund **15** (0.19 g, 0.29 mM) was dissolved in 50 mL of hydrochloric acid 37% (HCl). $SnCl_2$ (0.52 g, 2.29 mM) was added in 4 small portions and the solution was stirred for 1h at 65°C. The reaction was quenched by ice (250 g), pH was set to pH 8 by diluting with 33% NH_3 and the product was extracted 4 times by DCM. The organic fractions were collected, dried over $MgSO_4$ and purified by column chromatography (silica gel, DCM as eluent) yielding the product as a dark-green powder (0.026 g, 19%). ^{1}H NMR (500 MHz, DMSO-d_6) δ: -2.86 (2H, s), 4.35 (2H, s), 7.02 (2H, J = 8.4 Hz, d), 7.86 (9H, m), 8.23 (8H, m), 8.81 (6H, s), 8.97 (2H, s). ESI-MS. m/z: 630 (M-H$^+$).

Synthesis of 5-(1,2-dithiolan-3-yl)-N-(4-(10,15,20-triphenylporphyrin-5-yl)phenyl)pentanamide (12). The compound **16** (0.01 g, 0.016 mM), triethyleneamine (0.009 mL, 0.063 mM) and HOBt (0.008 g, 0.063 mM) were dissolved in 25 mL of anhydrous DMF under N_2 atmosphere and stirred at 0°C for 15min. Subsequently, EDC (0.012 g, 0.063 mM) was added and stirred for 15 min. Lipoic acid (0.013 g, 0.063 mM) was added and the solution was maintained overnight at 50°C. The mixture was concentrated under reduced pressure and purified by column chromatography (silica gel, DCM as eluent) yielding the product as a dark-green powder (0.18 g, 60%). ^{1}H NMR (500 MHz, DMSO-*d*$_6$) δ: 1.33–1.61 (6H, m), 1.66–1.94 (2H, m), 2.17–2.35 (2H, m), 2.91 (2H, s), 3.57–3.78 (1H, m), 5.24 (1H, s), 7.85 (9H, m), 8.07 (2H, J = 8.4 Hz, d), 8.23 (8H, m), 8.87 (6H, s), 8.90 (2H, s). ^{13}C NMR (126 MHz. Acetone-*d*$_6$) δ: 55.51, 59.39, 117.42, 120.17, 126.95, 127.95, 134.54, 141.96. MALDI TOF-TOF-MS: m/z: 818.4260 (M-H$^+$), 834.6216 (M-O$^+$), 850.7180 (M-O_2^+). UV-Vis: λ_{max} = 417 nm, 512 nm, 550 nm, 590 nm 646 nm in MeOH. Fluorescence: λ_{em} = 651, 716 nm in MeOH.

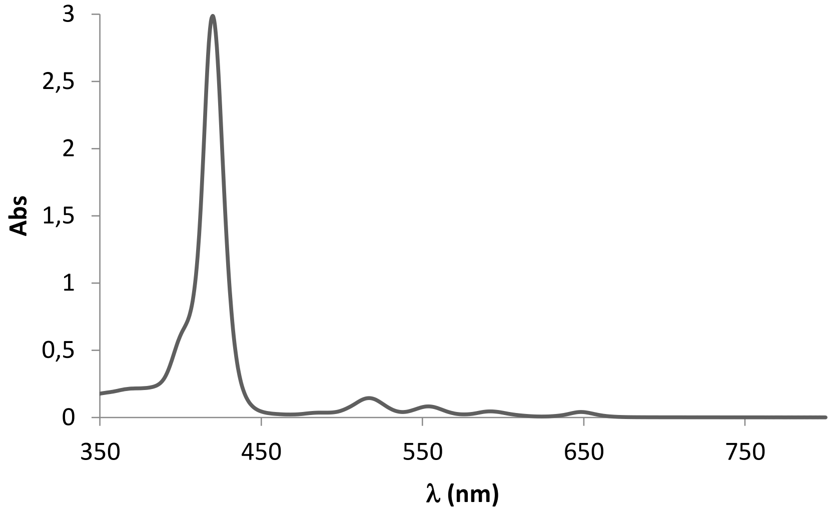

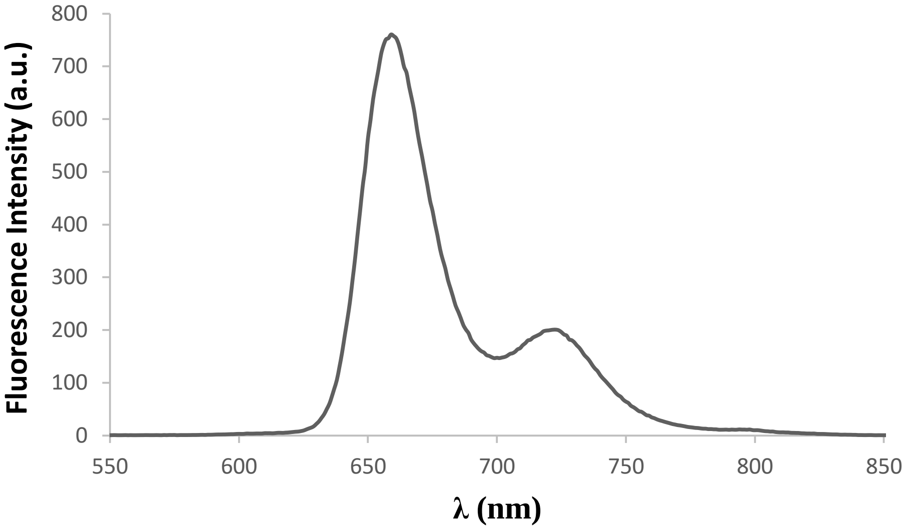

Figure S3: UV-VIS spectrum (top) and fluorescence spectrum (bottom) of compound **12** in MeOH**.**

3. General synthesis of poly-lipoic nanoparticles

The lipoic acid-based nanoparticles were synthetized combining the nanoprecipitation method with the ring-opening disulfide exchange polymerization.

An acetone solution of the precursor (20 mL, 5 mg/ml) –or of mixed precursors- was added dropwise with a syringe pump (0.5 ml/min) to an aqueous solution (100 mL) of Pluronic surfactant (100 mL, 20 mM) buffered at pH 7.4 with PBS (2 mM of 2 mM of phosphate buffer) under stirring (1000 rpm). After the addition of precursor, the solution was left stirring for 30 min. Next, 2 mL of a solution of 1-octanethiol (5 mg/ml) in acetone was added. The polymerization reaction was mantained for 90 min, then 2 mL of a iodoacetamide (5 mg/ml) solution in acetone was added to terminate the polymerization. The reaction mixture, now milky, was concentrated under reduced pressure and filtered with a 0.20 µm cutoff cellulose acetate filter. Nanoparticles were collected by centrifugation and washed one time by centrifugation in PBS (pH 7.4, 12 mL, 2 mM of phosphate buffer) and twice by centrifugation in H_2O (12 mL each). Final product was stored at $4^{o}C$ as water suspension.

The hydrodynamic particle size (Dynamic Light Scattering, DLS) and Z-potential were measured with a Malvern Zetasizer Nano-S equipped with a HeNe laser (633nm) and a Peltier thermostatic system. Measurements were performed at 25 °C in water or PBS 10 mM buffer at pH 7. Transmission electron microscopy (TEM) was recorded on a FEI Tecnai G12 microscope operating at 100 kV. The images were registered with a OSIS Veleta 4K camera. Thermogravimetric analysis (TGA) was run on 100 µl nanoparticle samples using a Q5000 IR instrument from 25 to 1000 °C under a continuous air flow.

The hydrodynamic particle size (Dynamic Light Scattering, DLS) and Z-potential were measured with a Malvern Zetasizer Nano-S equipped with a HeNe laser (633nm) and a Peltier thermostatic system. Measurements were performed at 25 °C in water or PBS 10 mM buffer at pH 7. Transmission electron microscopy (TEM) was recorded on a FEI Tecnai G12 microscope operating at 100 kV. The images were registered with a OSIS Veleta 4K camera. Thermogravimetric analysis (TGA) was run on 100 µl nanoparticle samples using a Q5000 IR instrument from 25 to 1000 °C under a continuous air flow.
NMR spectra in the solid state were collected on a Varian 400 equipped with a narrow bore, triple resonance T3 MAS probe spinning 4 mm rotors and operating at ^{1}H and^{13}C frequencies of 400.36 and100.68 MHz, respectively. The nominal temperature of the probe was always set to 298 K. ^{13}C CP-MAS spectra were acquired at 5 kHz MAS with 1200 scans and a repetition delay up to 3 s. The contact time for CP was 2 ms, and an acquisition time of 50 ms was used.

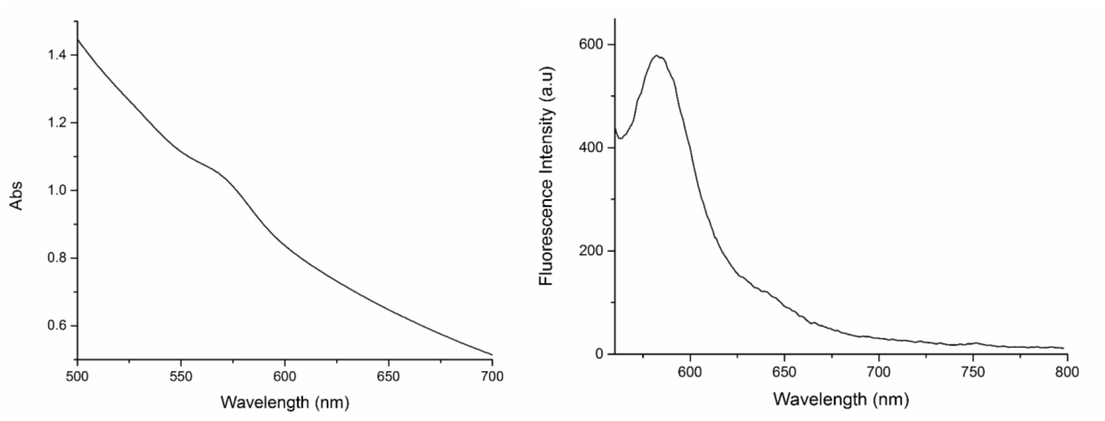

Figure S4: Absorption (left) and fluorescence intensity spectra (right) of nanoparticles made of 95% **7** and 5% of **10.**

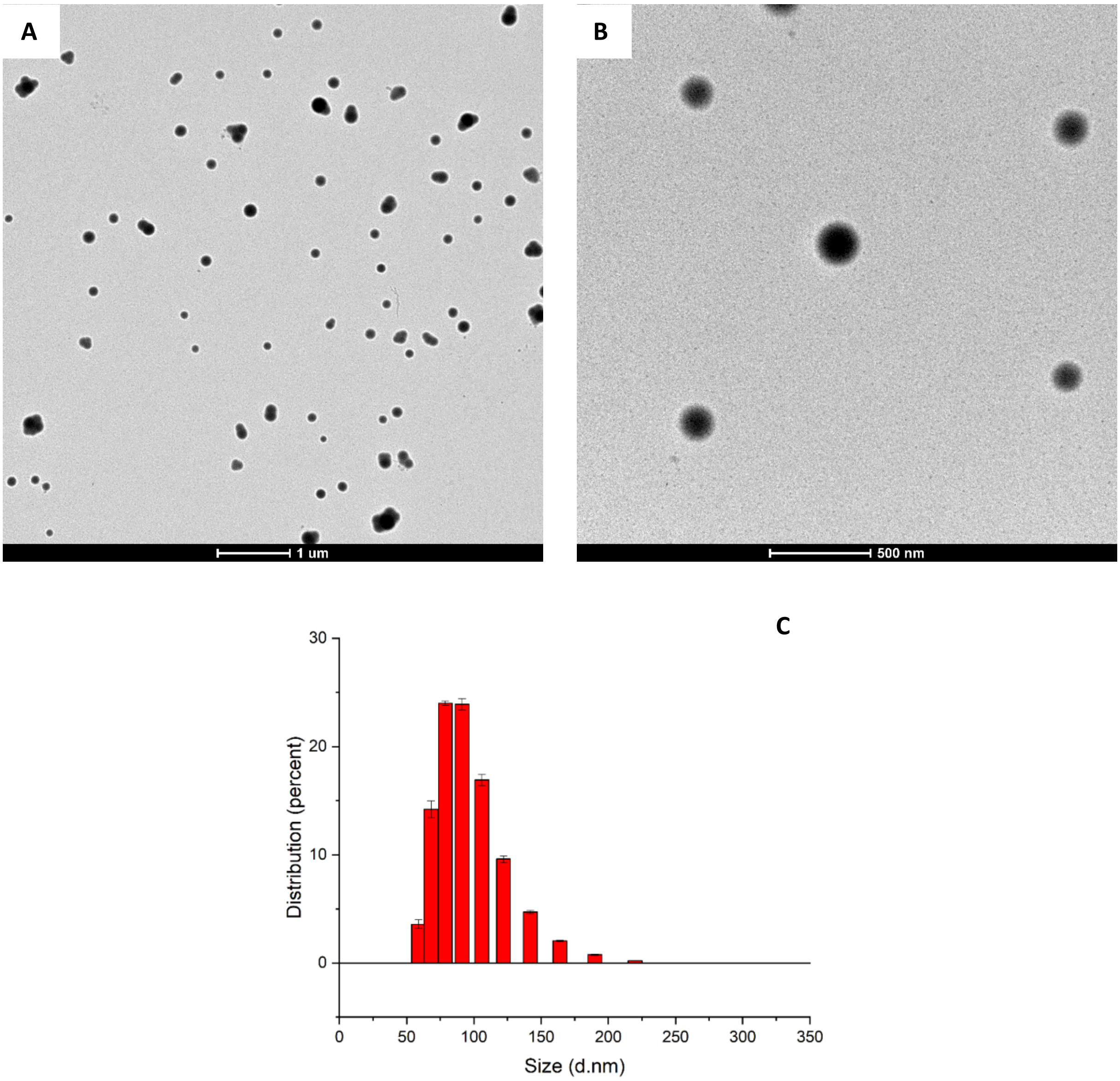

Figure S5: The TEM micrographs (A and B) and DLS histogram (C) of nanoparticles made of 95% **7** and 5% of **10.**

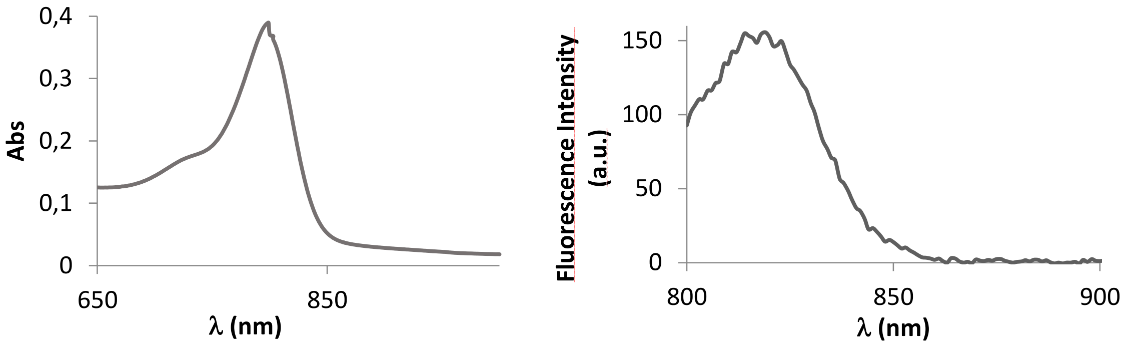

Figure S6: Absorption (top) and fluorescence intensity spectra (bottom) of nanoparticles made of 95% **7** and 5% of **11**.

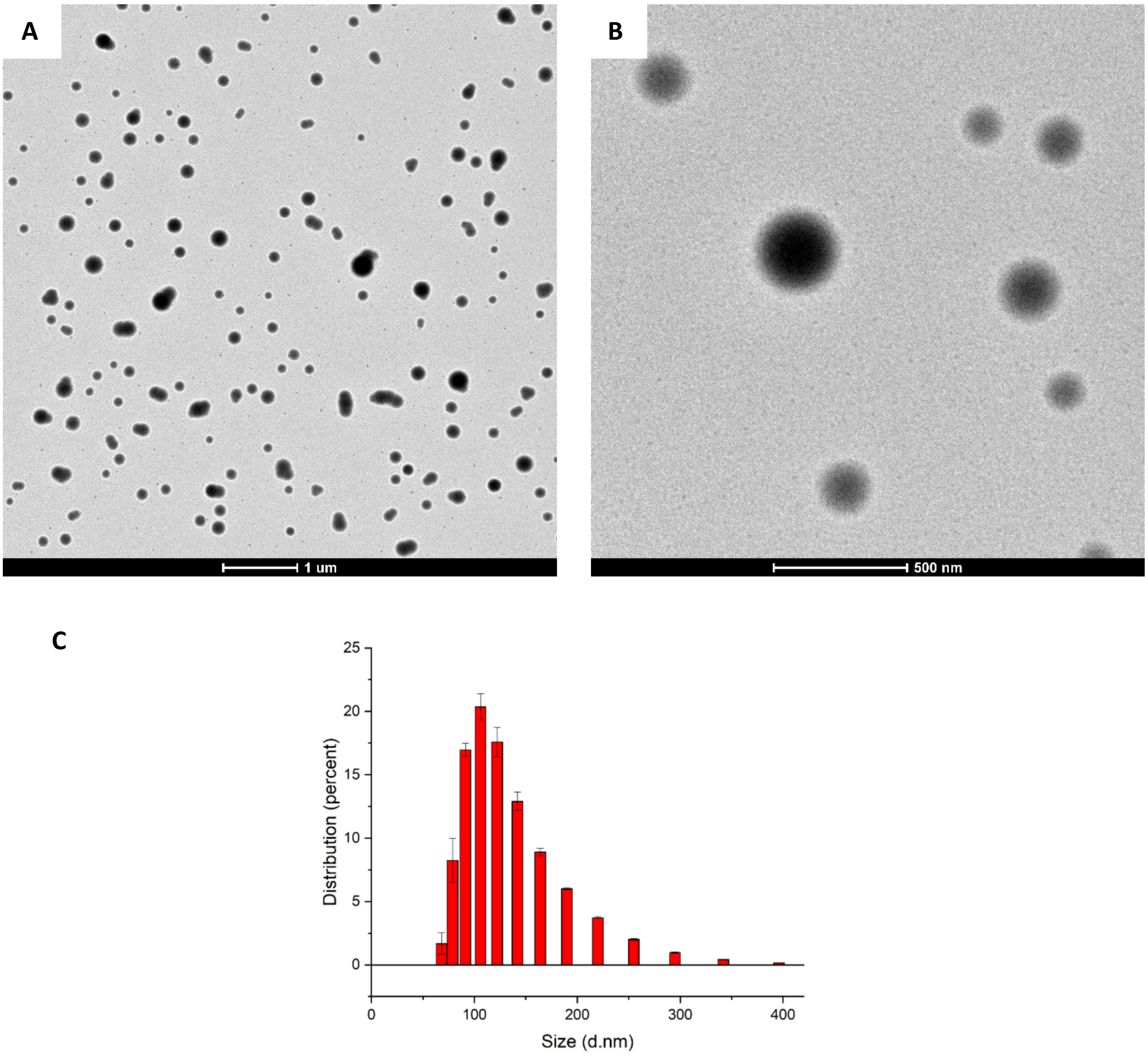

Figure S7: The TEM micrographs (A and B) and DLS histogram (C) of nanoparticles made of 95% **7** and 5% of **11**.

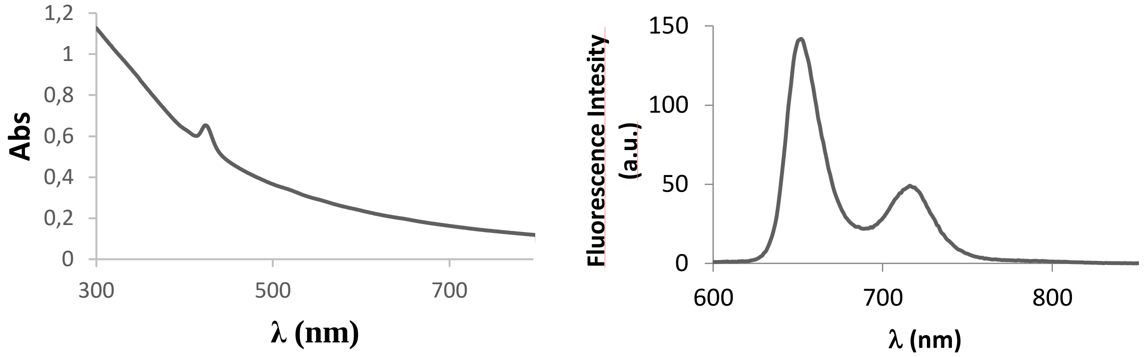

Figure S8: Absorption (top) and fluorescence intensity spectra (bottom) of nanoparticles made of 95% **7** and 5% of **12**.

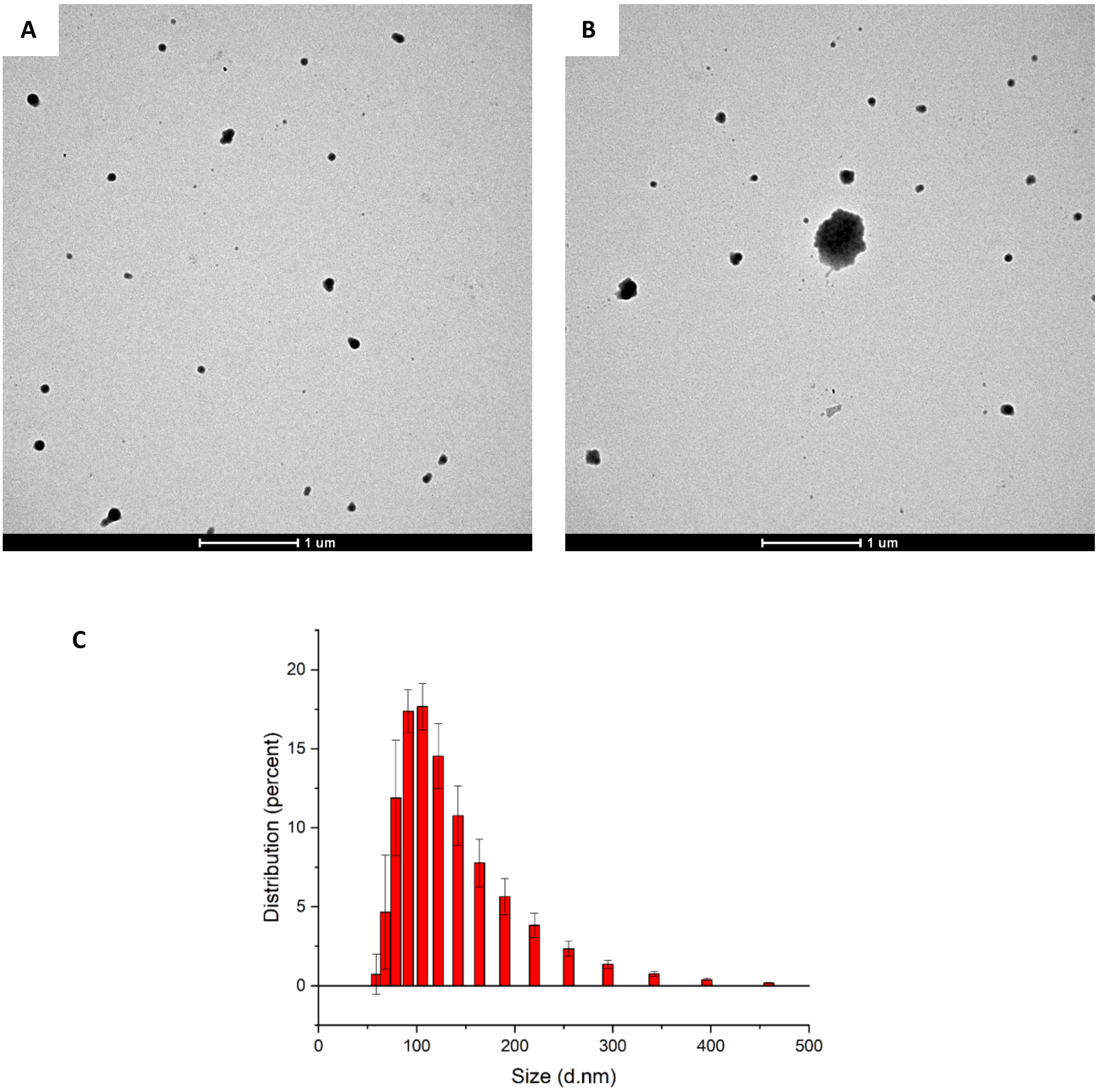

Figure S9: The TEM micrographs (A and B) and DLS histogram (C) of nanoparticles made of 95% **7** and 5% of **12.**

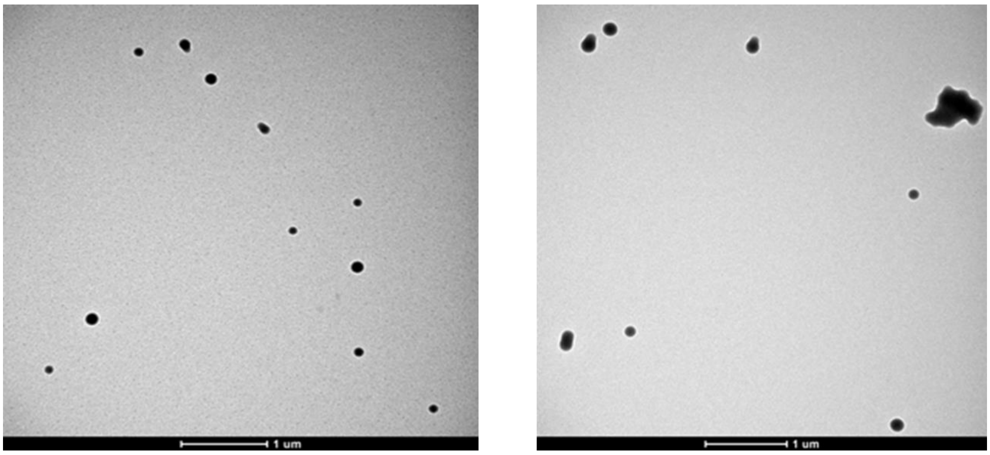

Figure S10: The TEM micrographs of nanoparticles made from precursor **7** using F-68 surfactant.

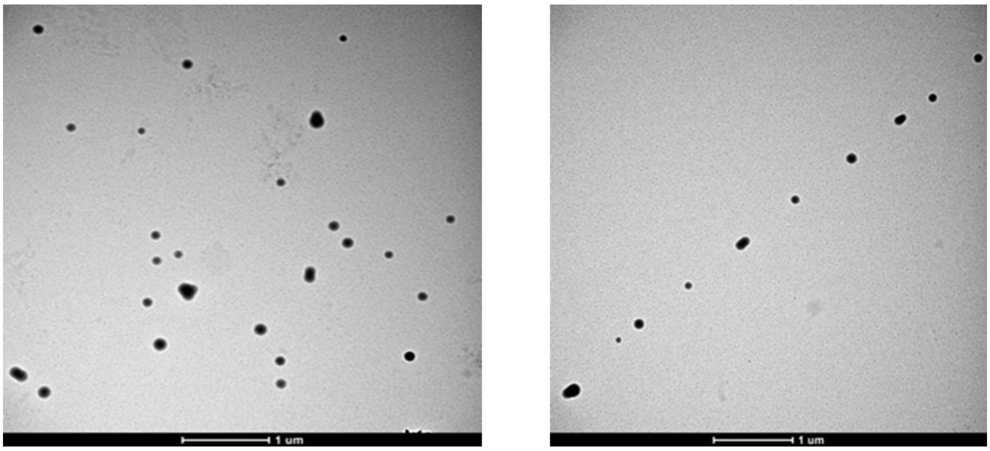

Figure S11: The TEM micrographs of nanoparticles made from precursor **7** using F-127 surfactant.

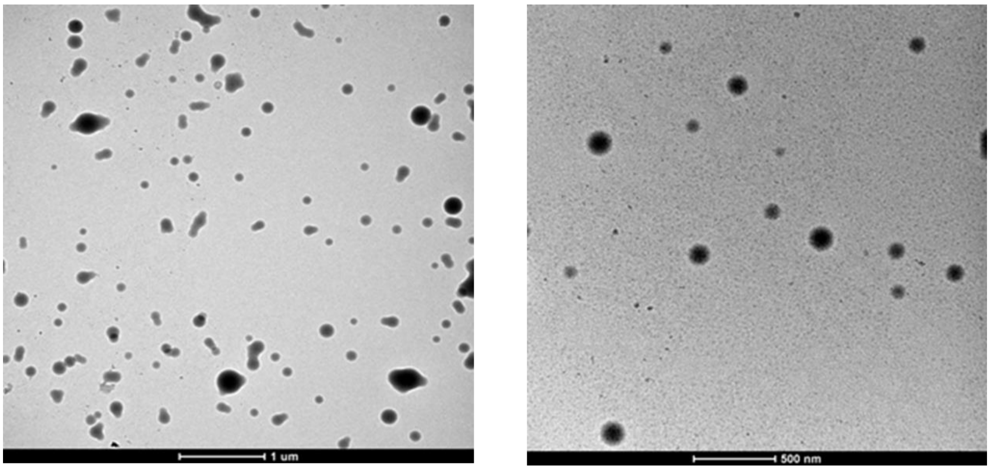

Figure S12: The TEM micrographs of nanoparticles made from precursor **8** using F-68 surfactant.

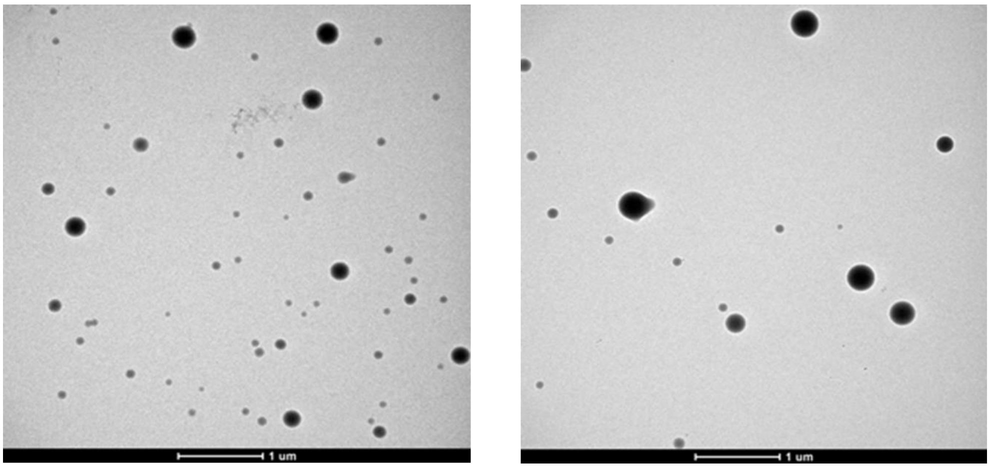

Figure S13: The TEM micrographs of nanoparticles made from precursor **8** using F-127 surfactant.

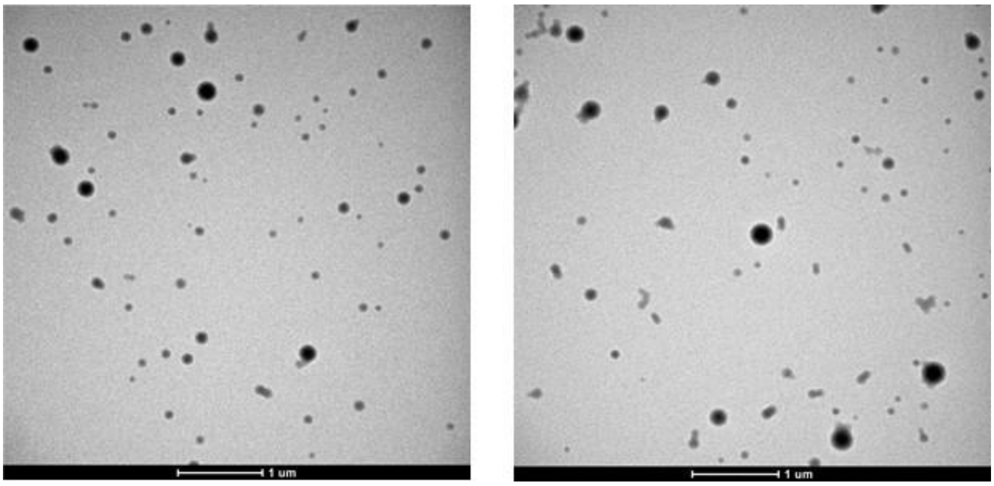

Figure S14: The TEM micrographs of nanoparticles made from precursor **5** using F-68 surfactant.

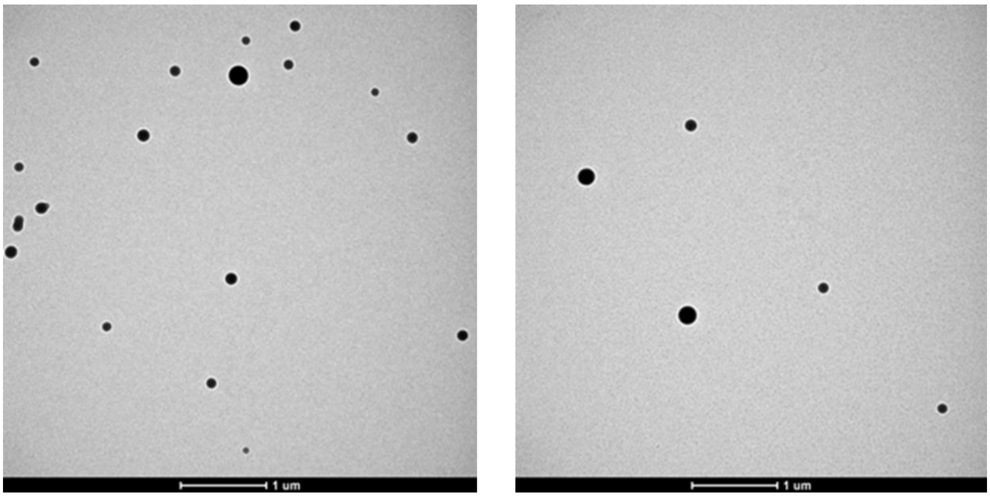

Figure S15: The TEM micrographs of nanoparticles made from precursor **5** using F-127 surfactant.

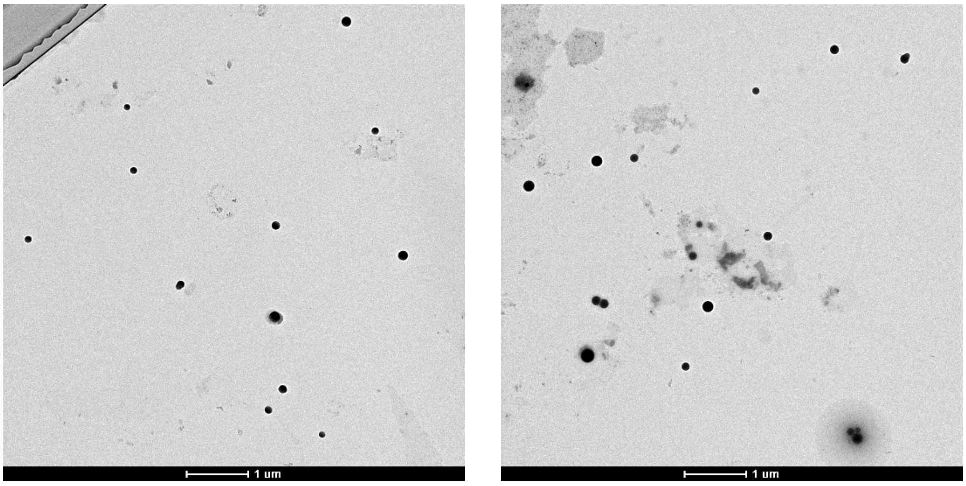

FigureS16: The TEM micrographs of nanoparticles made from precursor **9** using F-68 surfactant.

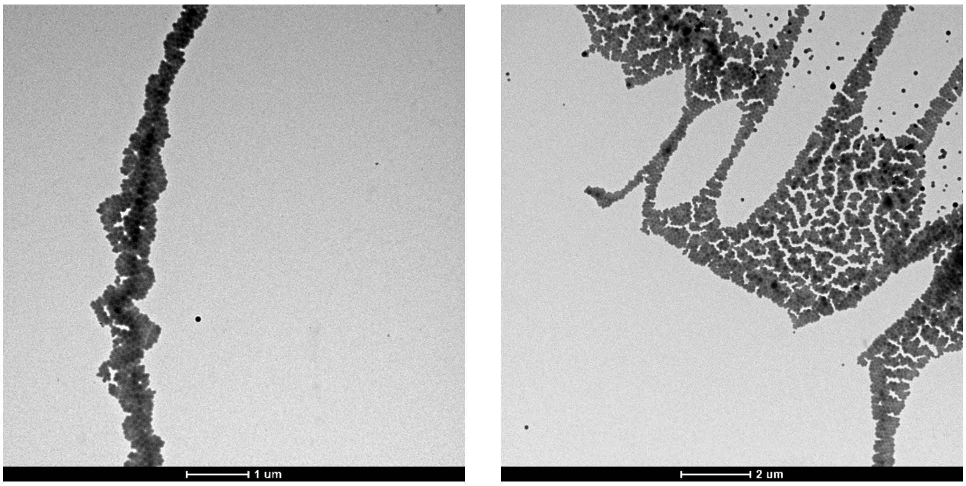

Figure S17: The TEM micrographs of nanoparticles made from precursor **9** using F-127 surfactant.

Synthesis of PLGA NPs

An acetone solution of a 35 KDa 50:50 PLGA-PEG_{5000} copolymer (20 mL, 5 mg/ml) was added dropwise with a syringe pump (0.5 ml/min) to an aqueous solution (100 mL) of Pluronic surfactant (100 mL, 20 mM) buffered at pH 7.4 with PBS (2 mM of 2 mM of phosphate buffer) under stirring (500 rpm). After the addition of precursor, the solution was left stirring for overnight. The reaction mixture was then collected by centrifugation and washed three times with H_2O (3 mL each). Final product was stored at 4°C as water suspension (average size 95 nm, PDI 0.27 measured by DLS).

4. Chemical characterization of nanoparticles

1. Effect of the addition of the RODEP initiator

F127@**7**-NPs were prepared with the procedure reported above but with and without initiator/terminator addition. Samples were stored in water at room temperature and the particles hydrodynamic diemter was monitered by DLS over 21 days (Table S2). TEM analysis was performed on both fresh and aged samples (Figure S18).

	Day 0	Day 3	Day 8	Day 12	Day 21
F127@**7**-NPs<br>Size (nm); PDI	160 ± 35.42;<br>0.049	177 ± 41.51;<br>0.055	189 ± 53.79;<br>0.081	191 ± 58.25;<br>0.093	182 ± 52.43;<br>0.083
F127@**7**-NPs<br>No initiator<br>Size (nm); PDI	172 ± 34.83;<br>0.041	205 ± 43.49;<br>0.045	243 ± 78.37;<br>0.104	243 ± 77.99;<br>0.103	304 ± 122.36;<br>0.162

Table S2: Summary table of DLS measurements of F127@**7**-NPs (top) and F127@**7**-NPs without activator/terminator (bottom).

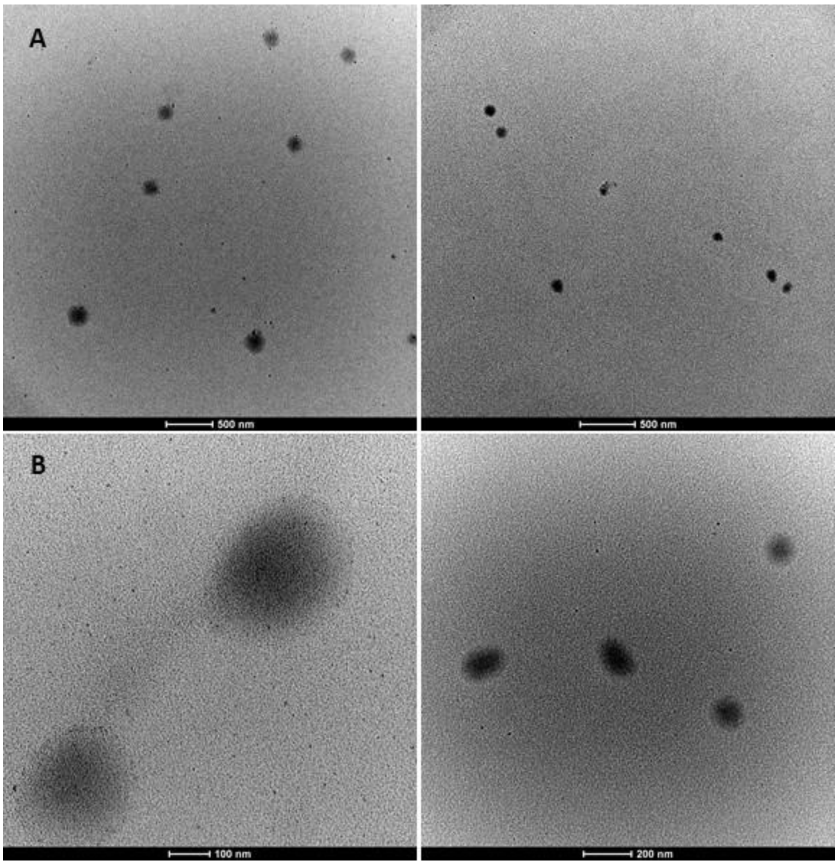

Figure S18: TEM images day 0 (left) and day 12 (right) of A) F127@**7**-NPs, and B) F127@**7**-NPs no activator/terminator.

2. Elemental analysis of nanoparticles

5 mg of F127@**7**-NPs and F127@**9**-NPs were lyophilized and analyzed by elemental analysis in triplicate from independent batches of NPs.

Considering the fraction of polymer molecules obtained by elemental analysis, the surface area of the NPs (extimated by assuming a density of the polymeric core of 0.6 mg/mL) and the fact that each F127 molecule bears two PEG chains, a polymer grafting density of 0.49 and 0.50 PEG chains nm^{-2} was calculated, for F127@**7** and F127@**9**, respectively.

These values corresponded to a distance between grafting points (D) of 1.60 and 1.61 nm, respectively, which, taking into consideration the 5.5 nm Flory radius (R_f) of PEG(4400), indicates that the polymer is in brush conformation ($D < R_f$). In addition, the length of the grafted PEG chains (L) can be estimated to be 12,7 nm. Since $L > 2R_f$ we can confidently conclude that both NP types feature a dense brush shell.[5]

Calculation were repeated with different core density values up to 1.2 g/cm^3, in all the case grafting densities obtained are in the range of dense brush shell.

	**Found elemental analysis 1 (%)**	**Found elemental analysis 2 (%)**	**Found elemental analysis 3 (%)**	**Calculated average weight percentage (%) of F127**	**Average footprint F127 Pluronic (nm^2)**
F127@**7**-NPs	C 54.53, H 8.65, S 18.41, O 18.41	C 54.11, H 8.26, S 18.69, O 18.94	C 54.30, H 8.42, S 20.04, O 17.24	22.62	4.11 ± 1.11
F127@**9**-NPs	C 48.87, H 7.48, S 18.12, O 25.53	C 49.02, H 7.18, S 23.01, O 20.79	C 49.01, H 7.01, S 21.77, O 22.21	28.50	4.04 ± 1.61

Table S3: Summary table of elemental analysis of F127@**7**-NPs and F127@**9**-NPs and their footprint.

3. Nanoparticles degradation

Samples of rhodamine (**10**) labelled F127@**7**-NPs and F127@**9**-NPs were incubated with 10 mM dithiothreitol (DTT) at 37°C, and the average size was monitored by DLS every hour. TEM analysis was performed on F127@**7**-NPs and F127@**9**-NPs upon incubation with DTT and GSH 1 mM and 10 mM at 37ºC for 1 hour.

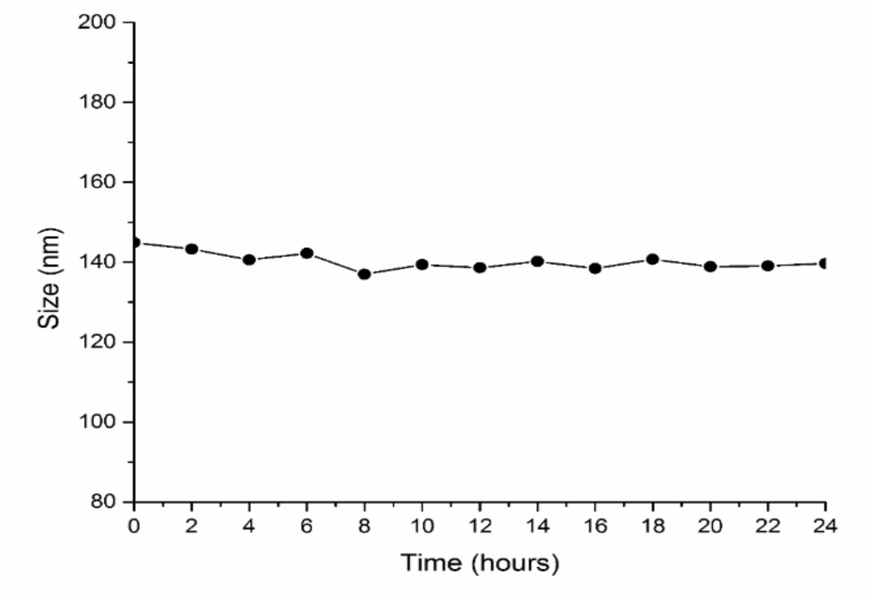

Figure S19: Time dependence of the size of rhodamine (**10**) labelled F127@**7**-NPs upon incubation with DTT 10 mM.

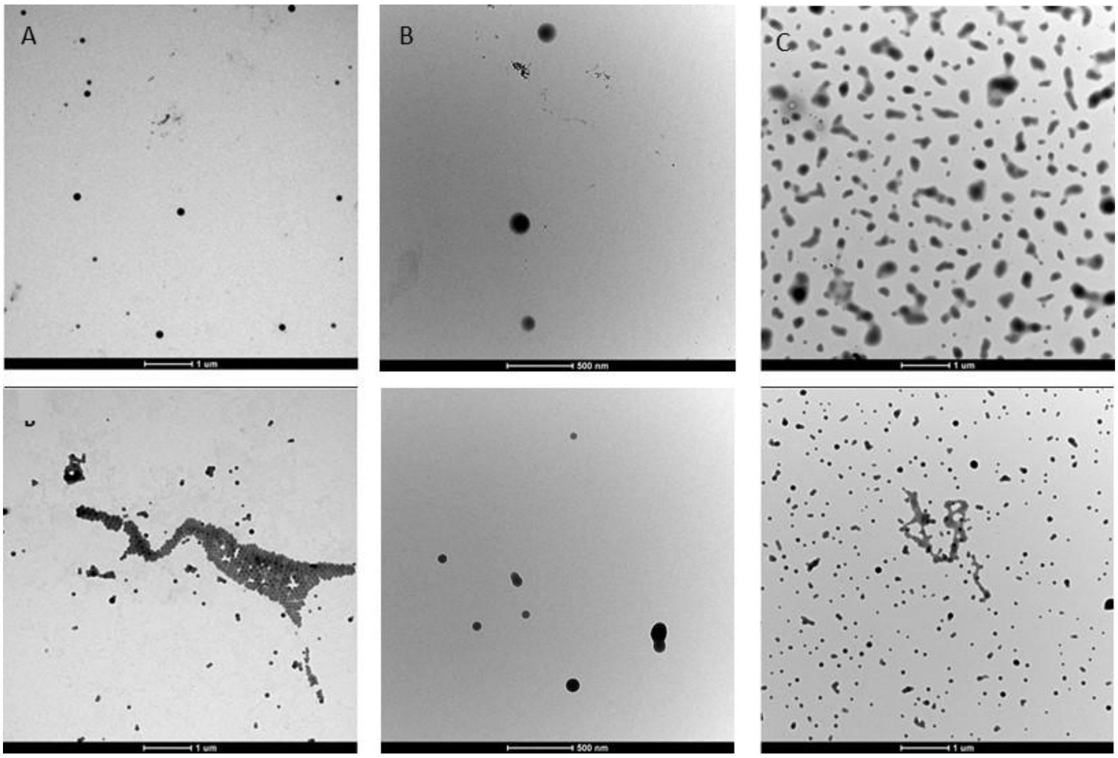

Figure S20: TEM images of F127@**7**-NPs (top) and F127@**9**-NPs (bottom) before addition of GSH (A), after 1h incubation with GSH 1mM (B) or GSH 10mM (C).

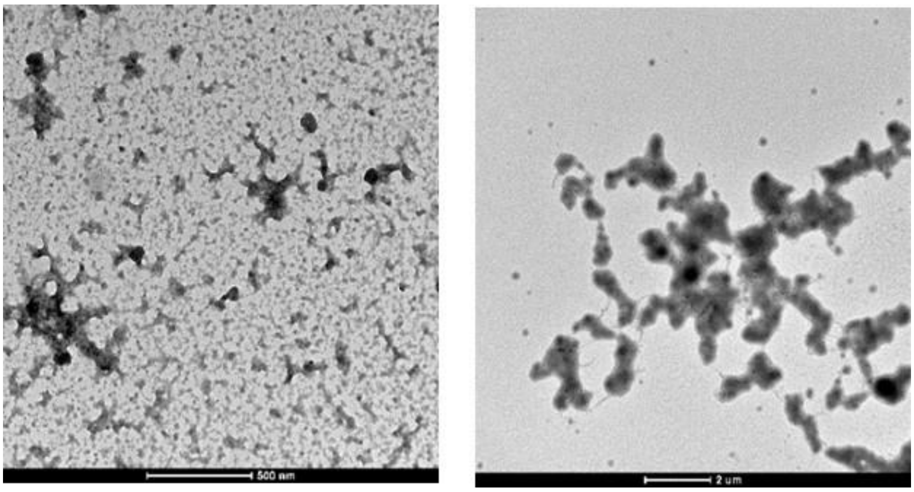

Figure S21: TEM images of F127@**7**-NPs incubated at 37 °C for 24h in 10 mM glutathione (left) and 10 mM DTT (right).

Samples of rhodamine (**10**) labelled F127@**7**-NPs and F127@**9**-NPs were incubated for 1 h at 37°C with GSH 1 mM and 10 mM, the nanoparticles were then recovered by centrifugation and resuspended in water. Residual 10 content was determined by the absorbance of 10 at 561 nm.

	F127@**7**	F127@**7**
Initial	14.0 μM	36.2 μM
GSH 10mM	12.3 μM (decrease of 12.1%)	33.2 μM (decrease of 8.3%)
GSH 1mM	13.8 μM (decrease of 1.4 %)	35.3 μM (decrease of 2.5%)

Table S4: Concentration of dye 10 in F127@**7**-NPs and F127@**9**-NPs samples before and after 1 h incubation at 37 °C with 1 and 10 mM GSH.

4. General procedure for nanoparticles freeze-drying

50 µL of a F127@**7** NPs stock solution (10 mg/ml) doped with **10** (5% with respect to **7**, average size 84 nm, PDI: 0,08 measured by DLS) were diluted to 1,5 mL with a trehalose solution (5%, 10%, 25% and 30% w/v) in H_2O and frozen in liquid N_2 for 1 hour. Afterwards, vials were placed in separated round bottomed flasks, covered with aluminum film and lyophilized overnight. Subsequently, lyophylates were resuspended in H_2O (1,5 ml) by sonication for 10 min and analyzed via DLS.

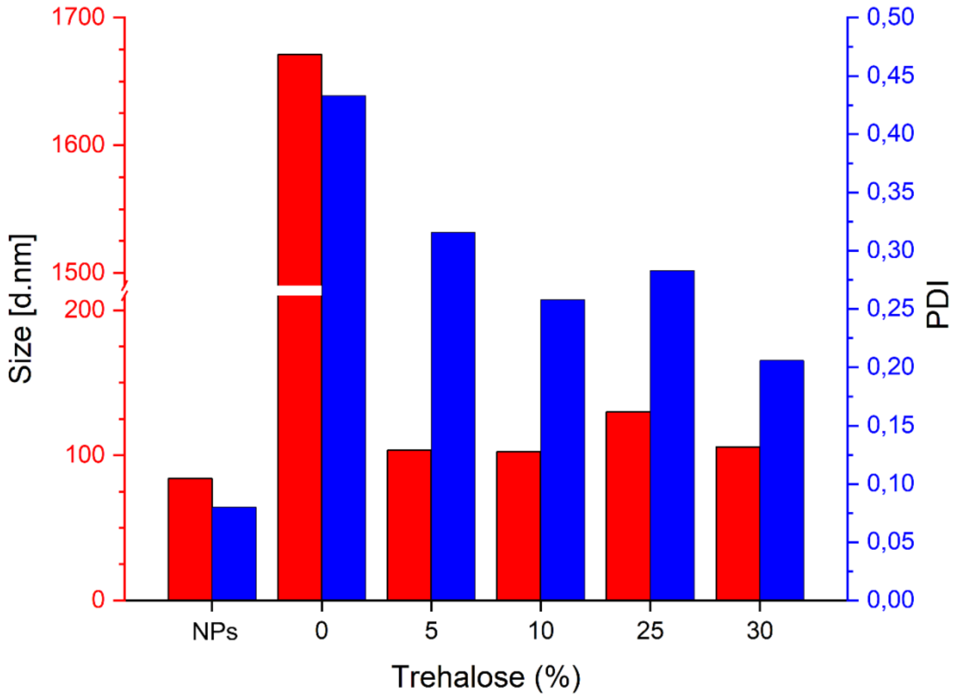

Figure S22:. Size [d.nm] (red) and PDI (blue) changes upon NPs lyophilization with the presence of different trehalose solutions. NPs stands for 50 µl of NPs stock solution (10 mg/ml) diluted to 1,5 mL with H_2O without freeze-drying.

5. In vitro characterization of nanoparticles

1. Serum or plasma proteins associated to NPs

NPs (50 µg/mL) were incubated at 37°C for 30' with 10%, 20% or 50% HS (or HP or FCS) diluted in RPMI-1640 medium, recovered by centrifugation (30 minutes, 12000 rpm at 4°C), washed one or three times with PBS and dissolved in 25 µL of loading sample buffer. NP pellets were heated at 95°C for 5 min and loaded in equal volumes (12 µL) on a 12% (v/v) SDS-PAGE. Proteins were stained with Silver Staining protocol or blotted onto PVDF membrane (Amersham) and HRG, HSA or Apo A1 were detected by specific antibodies (Abnova for HRG, Calbiochem for HSS and Apo A1) by enhanced chemi-luminescence reaction.

2. Plasma clotting time

77 µL of HP were added to 100 µL of NPs or Ludox® at various concentrations in 150 mM NaCl in a 96-well microtiter plate (Sarstedt) and coagulation was started by the addition of 23 µL of 150 mM $CaCl_2$. The plate was incubated at 37°C and the changes in optic density were read at 405 nm every 60s 60 times. To calculate the mean absorbance at each time point, three wells were averaged per sample. The time required to reach half maximal absorbance increase (t½) was calculated and used for statistical analysis.

3. C3a detection

To control complement activity, 25 µL of HS were treated with 6.25 µL of zymosan (25 mg/mL, Sigma, prepared as described by manufacturer's instructions) for 30 minutes at 37°C; reaction was stopped with 25 mM EDTA. To assess complement activation of HS induced by NP incubation, 25 µL of HS were incubated with different concentrations of NPs for 30 minutes at 37°C. Then 1.6 µL of each sample were mixed with 38.4 µL of water and 6.7 µL of loading sample buffer and 15 µL of sample were loaded onto a 12% gel. Proteins were then blotted onto PVDF membrane and C3a was detected by specific antibodies (Cabiochem) by enhanced chemi-luminescence reaction.

4. Hemolysis assay

Human erythrocytes were obtained from human blood of healthy volunteers after elimination of buffy coats, washed in PBS, and further treated with different NP doses (up to 100 µg/mL) in triplicate; after 2 h incubation, samples were centrifuged (1500 rpm for 5 min) and supernatant absorbance determined at 540 nm; data were expressed as percentage with respect to positive control (human erythrocytes incubated with water).

5. Cells

HeLa and Raw 264.7 cells were maintained in DMEM (Invitrogen), supplemented with 10% FCS (Euroclone) and antibiotics (penicillin and streptomycin, Invitrogen) at 37 °C in a humidified atmosphere containing 5% (v/v) CO_2; cells were split every 2–3 days. Human macrophages were obtained from human monocytes, purified from buffy coats of healthy donors by means of two sequential centrifugations on Ficoll and Percoll (GE Healthcare) gradients and differentiated for 7 days with 100 ng/mL macrophage colony-stimulating factor (M-CSF, BD Biosciences) in RPMI-1640 plus 20% FCS. Total human leukocytes were obtained from buffy coats after erythrocytes lysis by hypotonic shock in 155 mM NH_4Cl, 10 mM $KHCO_3$ and 100 mM Na_2EDTA at pH 7.4 for 3 min at room temperature.

6. MTT assay

The day before the experiment cells (HeLa, human leukocytes, human macrophages and mouse Raw 264.7) were seeded onto 24 well plate (Falcon) as indicated in Figure 8 (B and C). The day of the experiment cells were treated with different concentrations of NPs (up to 100 µg/mL, as indicated in Figure 8) in cellular medium supplement with 10% FCS; after 24 h or 6 days incubation, wells were sucked off and incubated with 100 µl of MTT (Promega) at 37°C until colour development; absorbance was red at 492 nm and the percentage of alive cells was calculated with respect to non-treated cells.

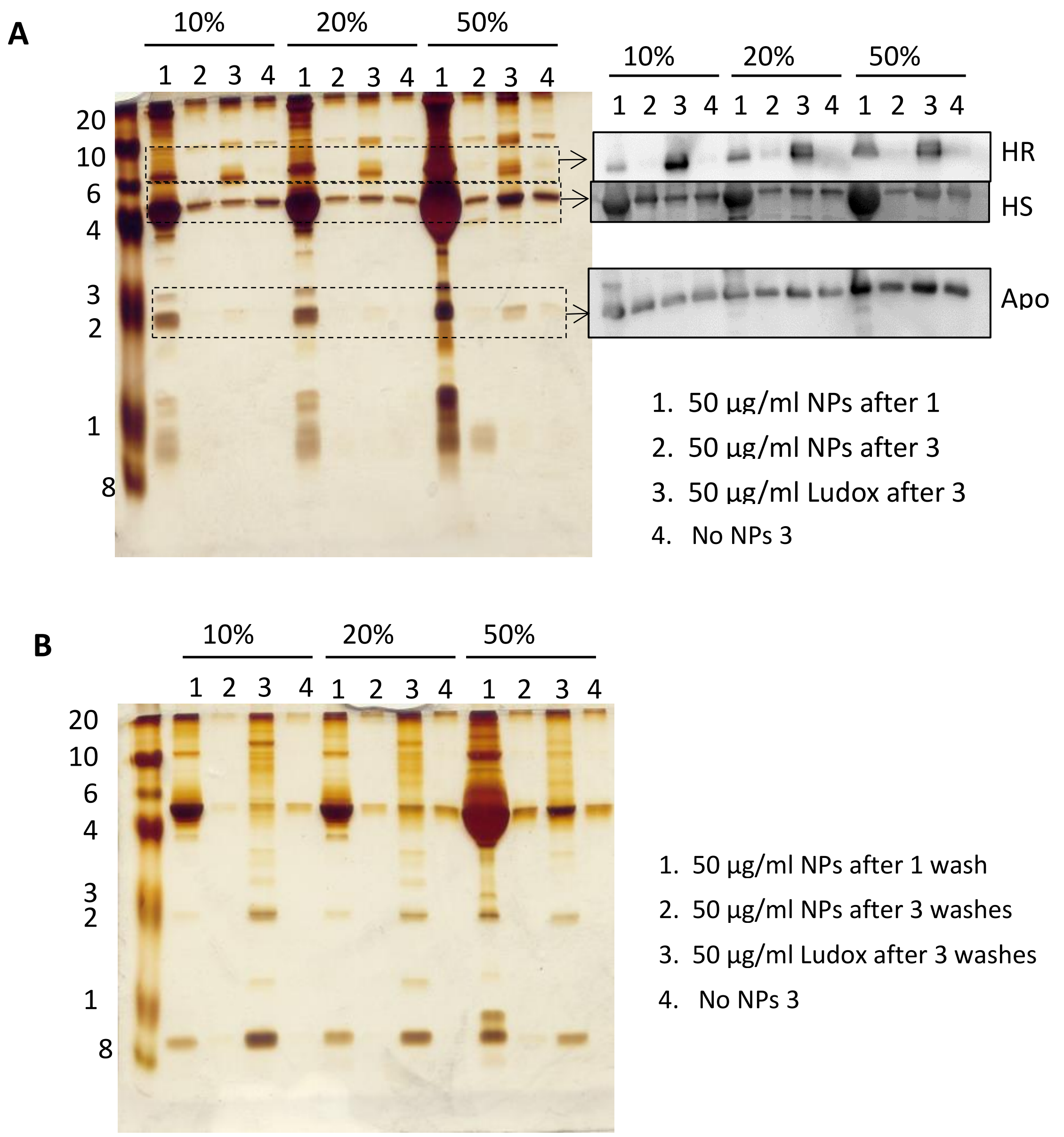

Figure S23: A) Silver staining (left panel) and western blot analysis (right panel) of proteins associated to NPs or Ludox® after 30 minutes incubation at 37°C with different concentrations of HP. B) Silver staining of proteins associated to NPs or Ludox® after 30 minutes incubation at 37°C with different concentrations of FCS.

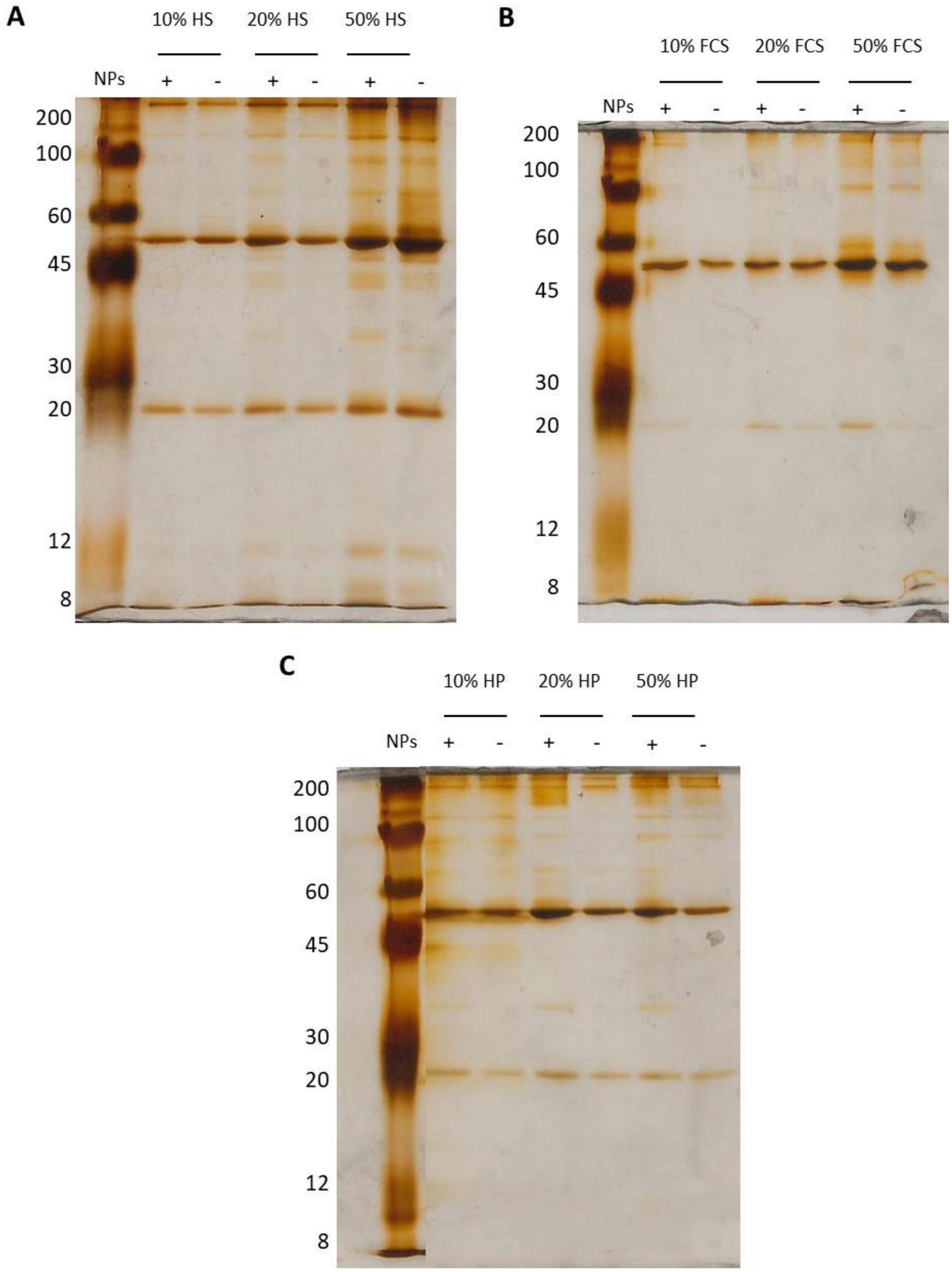

Figure S24: Silver staining of proteins associated to 50 µg/mL of PLGA-NPs after 30 minutes incubation at 37°C with different concentrations of HS (A), FCS (B) or HP (C); samples were washed three times in PBS.

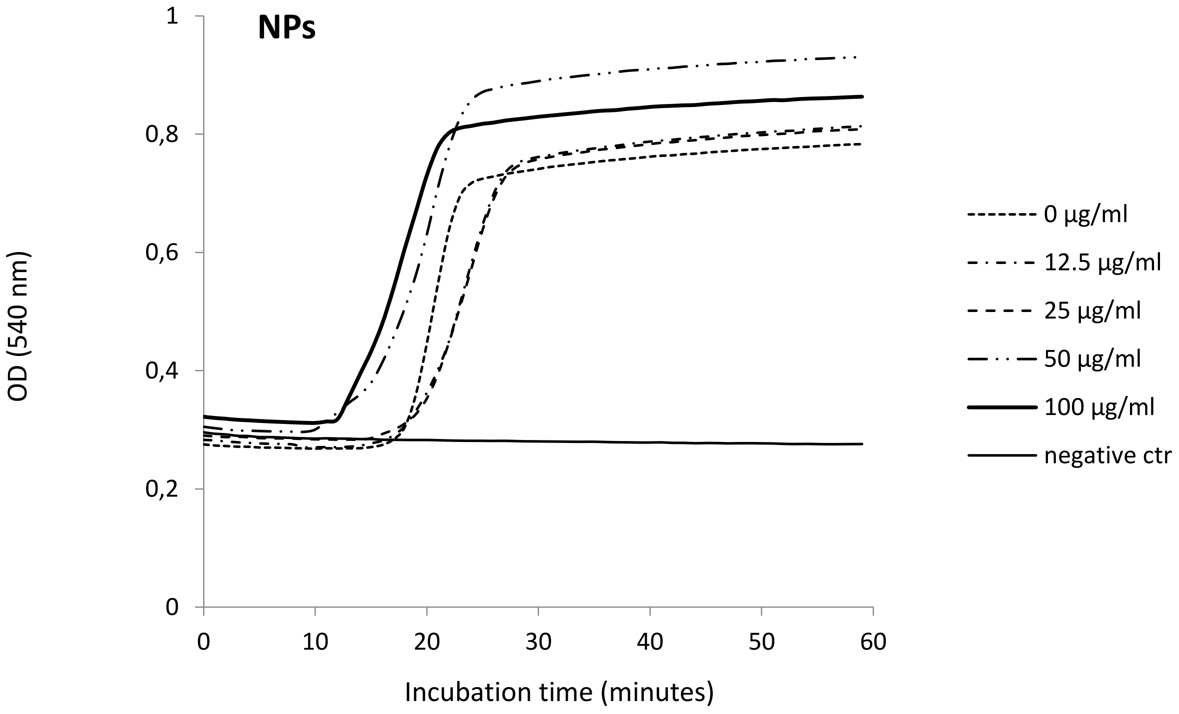

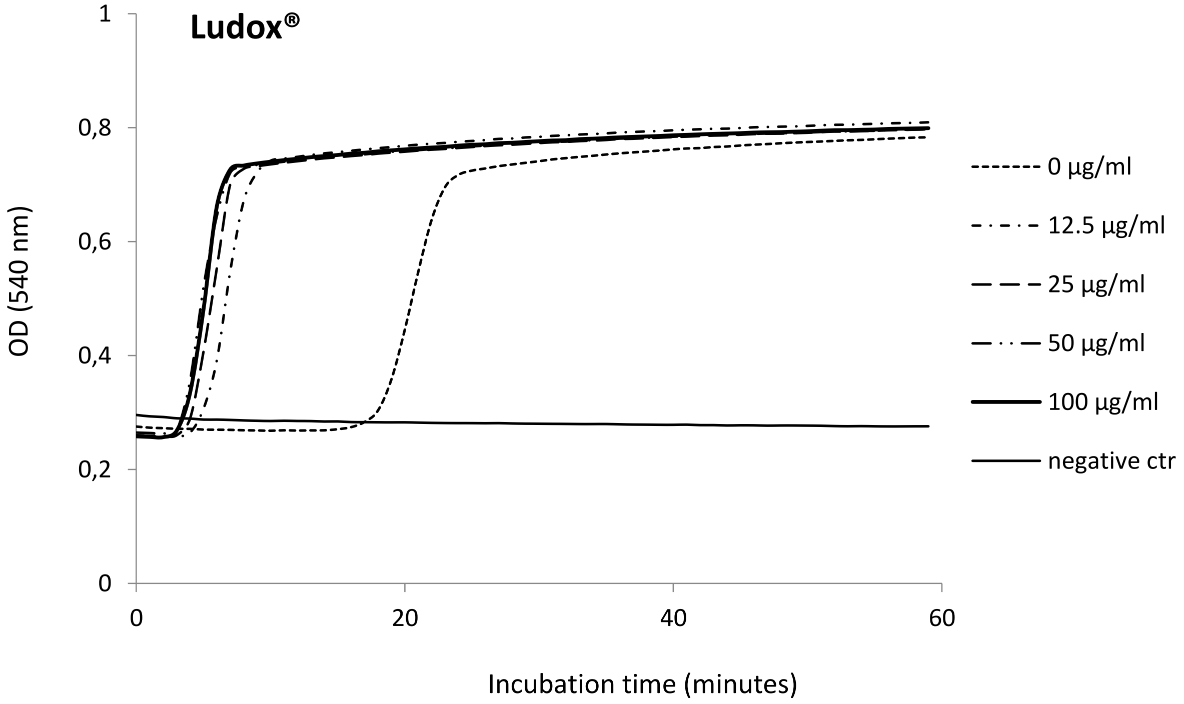

Figure S25: Representative coagulation kinetics of HP in the presence of the indicated types of NPs at different concentrations. Negative control (ctr) corresponds to the kinetics of HP in the absence of $CaCl_2$ and without additional stimuli.

6. In vivo characterization of nanoparticles

1. Animal model

Health male Sprague-Dawley rats, weighing 200 g, were injected with 2 mg/rat of rhodamine red labelled F127@**7**-NPs via tail vein. Rats were housed in a temperature-controlled environment (21–22 °C) on a 12 h light/dark cycle, with access to water and food at all times.

At different time points (T0, 1hour, 3hours, 1day, 3days, 7days and 1/2months) from injection, rats were randomly killed and blood and organs were collected. Organs have been removed in toto and washed in distilled water and fixed in formalin to reduce passive dissemination on the cut surface.

Experiments were approved by the University of Padua Ethical Committee and from Italian National Health Institute.

2. Neutrophil gelatinase-associated lipocalin (NGAL) assessment on sera

Neutrophil gelatinase-associated lipocalin (NGAL) was measured on sera with an enzyme-linked immunoassay (Rat NGAL ELISA kit; BIOPORTO Diagnostics, Bioexe Research Technology, Verona, Italy) following the manufacturer's instructions. The antibody was specific for rat NGAL.[6]

3. NGAL expression in the Heart tissue

Heart samples were homogenized and solubilized in sodium dodecyl sulphate (SDS) buffer.[7] Protein quantification was performed using Qubit® Proteinassay Kit (Life Technologies, Monza, Italy) according to the manufacturer's instructions. Protein samples were mixed with a non-reducing and reducing buffer and incubated for 5 min at either room temperature (reducing and non denaturating conditions) or 95oC (reducing and denaturating conditions).

All samples were subsequently separated on a 10% gel in a SDS-PAGE and transferred onto a nitrocellulose membrane (Amersham, Euroclone, Italy). The membrane was blocked for 1 h with 5% non-fat milk in the TBS containing 0.5% (v/v) Triton X-100. (Sigma-Aldrich), and incubated overnight with polyclonal goat antibodies against NGAL (1:500, Abcam, Prodotti Gianni, Milan, Italy). Blots were developed using the Super Signal West Femto ECL substrate (Pierce, Euroclone, Italy).

Blot image acquisition was performed using Alliance 2.7 (UVITEC, Eppendorf, Italy) and software Alliance 2.7 1D fully automated.[6]

4. Organs biodistribution of NPs

Slices of about 3-5mm thickness of different organs were introduce in the Alliance 2.7 (UVITEC, Eppendorf, Italy). Rhodamine red labelled F127@7-NPs fluorescence was obtained under chroma Alliance 2.7 and was analyzed by alliance 3D software (UVITEC, Eppendorf, Italy). The macrodistribution analysis of NPs fluorescence signal was obtain with the same gain and setup of the

image analyser. The intensity of fluorescence is represented, normalized, as pseudocolor scale bar that is consistent for all images and that is reported in the Figure 10A.

5. Laser scanning confocal microscopy analysis and NP localization

Fixed rat organs were dehydrated with ethanol, cleaned with xylene, embedded in paraffin and sliced into 5 μm sections. One section for each organ was counterstain with TO-PRO-3 for nuclei identification (Invitrogen, Molecular Probes, Eugene, OR) following standard procedures.[8] Micrographs were taken using a laser scanner confocal microscope (Model TCS-SL; Leica, Germany) equipped with beam splitter 488, 543 and 605 nm and FW TD 488/543/633 beam splitting excitation mirrors. Laser scanning confocal microscopy analysis was performed keeping acquisition parameters (laser power, aperture width, opening percentage and gain) constant.

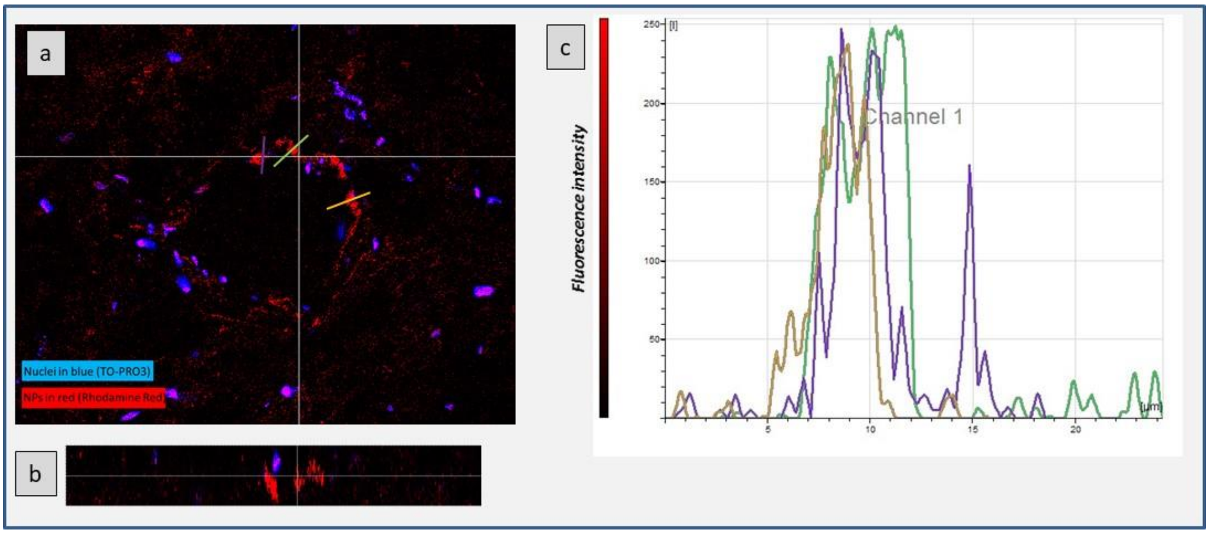

Figure S26: NPs localization. a) representative confocal laser microscopy image shows a vessel in the heart 1h after injection. Note as NPs are not invading the lumen of the vessel and are present in the heart interstitium and in the vessel wall. Nuclei are counterstain with TO-PRO3 (in blue) and NPs-rhodamine red conjugated in red. Zoom from original magnification 40x ; b) Z-stack view in the point where the grey lines cross in Figure a), showing the NPs distribution in all thickness of the section showing that NPs are able to localized in the depth of the tissue; c) Profile energy of NPs. Fluorescence intensity histogram shows the fluorescence emission spectrum of NPs of ROI (yellow, green and purple bar respectively) in a).

7. References

1. Lee, B. S.; Yuan, X.; Xu, Q.; McLafferty, F. S.; Petersen, B. A.; Collette, J. C.; Black, K. L.; Yu, J. S. Preparation and characterization of antioxidant nanospheres from multiple alpha-lipoic acid-containing compounds. *Bioorg. Med. Chem. Lett.*, **2009**, *19*, 1678-1681.
2. Nguyen, T.; Francis, B. M. Practical synthetic route to functionalized rhodamine dyes. *Org. Lett.*, **2003**, *5*, 3245-3248.
3. Strekowski, L.; Lipowska, M.; Patonay, G. Substitution reactions of a nucleofugal group in heptamethine cyanine dyes. Synthesis of an isothiocyanato derivative for labeling of proteins with a near-infrared chromophore. *J. Org. Chem*., **1992**, *57*, 4578-4580.
4. Luguya, R.; Jaquinod, L.; Fronczek, F. R.; Vicente, M. G. H.; Smith, K. M. Synthesis and reactions of meso-(p-nitrophenyl)porphyrins. *Tetrahedron*, **2004**, *60*, 2757-2763.
5. Perry, J. L.; Reuter, K. G.; Kai, M. P.; Herlihy, K. P.; Jones, S. W.; Luft, J. C.; Napier, M.; Bear, J. E.; DeSimone, J. M. PEGylated PRINT Nanoparticles: The Impact of PEG Density on Protein Binding, Macrophage Association, Biodistribution, and Pharmacokinetics. *Nano Lett*., **2012**, *12*, 5304-5310.
6. Angelini, A.; Castellani, C.; Virzì, G.M.; Fedrigo, M.; Thiene, G.; Valente, M.; Ronco, C.; Vescovo, G. The Role of Congestion in Cardiorenal Syndrome Type 2: New Pathophysiological Insights into an Experimental Model of Heart Failure. *Cardiorenal Med*., **2015**, *6*, 61-72.
7. Castellani, C.; Vescovo, G.; Ravara, B.; Franzin, C.; Pozzobon, M.; Tavano, R.; Gorza, L.; Papini, E.; Vettor, R.; De Coppi, P.; Thiene, G.; Angelini, A. The contribution of stem cell therapy to skeletal muscle remodeling in heart failure. *Int. J. Cardiol*., **2013**, *168*, 2014-2021.
8. Castellani, C.; Padalino, M.; China, P.; Fedrigo, M.; Frescura, C.; Milanesi, O.; Stellin, G.; Thiene, G.; Angelini, A. Bone-marrow–derived CXCR4-positive tissue-committed stem cell recruitment in human right ventricular remodeling. *Hum. Pathol*., **2010**, *41*, 1566-1576.
